# Development, Feasibility, and Appreciation of the Collaborative Integrated Depression Care (IDECA) Project in Flanders, Belgium

**DOI:** 10.3390/jcm15062326

**Published:** 2026-03-18

**Authors:** Ruben Willems, Kris Van den Broeck, Reini Haverals, Lieven Annemans, Pauline Boeckxstaens, Didier Schrijvers, Geert Goderis, Elke Peeters, Liesbeth Borgermans

**Affiliations:** 1 Interuniversity Center for Health Economic Research (I-CHER), Unit of Health Economics and Management, Department of Public Health and Primary Care, Ghent University, 9000 Ghent, Belgium; lieven.annemans@ugent.be (L.A.); liesbeth.borgermans@ugent.be (L.B.); 2 Department of Family Medicine and Population Health, University of Antwerp, 2000 Antwerp, Belgium; kris.vandenbroeck@uantwerpen.be; 3 Unit of Family Medicine, Department of Public Health and Primary Care, Ghent University, 9000 Ghent, Belgium; reini.haverals@ugent.be (R.H.); pauline.boeckxstaens@ugent.be (P.B.); 4 Department of Psychiatry, Collaborative Antwerp Psychiatric Research Institute (CAPRI) and Antwerp University Hospital, Faculty of Medicine and Health Sciences, University of Antwerp, 2000 Antwerp, Belgium; didier.schrijvers@uantwerpen.be; 5 Department of Psychiatry, University Psychiatric Center Duffel, 2570 Duffel, Belgium; 6 Department of Public Health and Primary Care, KU Leuven, 3000 Leuven, Belgium; geert.goderis@kuleuven.be; 7 Department of Medical Affairs, Johnson & Johnson, 2340 Beerse, Belgium; epeeter9@its.jnj.com

**Keywords:** depression, mental wellbeing, integrated care, case management, primary care

## Abstract

**Background**: Depression remains a major global health burden, yet fragmented care often leads to waiting times and unmet needs. Therefore, the Belgian collaborative Integrated Depression Care (IDECA) project strengthened primary care depression management by introducing a Reference Person Mental Wellbeing (RPMW) who functions as a case manager, supported by shared-care tools, structured psychoeducation modules, and targeted training for general practitioners (GPs). This study examines normalization in primary care practice. **Methods**: A single-arm, mixed-method study was implemented over 18 months in two Flemish Primary Care Zones (PCZ). Implementation outcomes were assessed every four months using the NoMAD questionnaire and analyzed using Wilcoxon signed-rank tests. Peer review sessions with professionals and interviews with patients were analyzed thematically. Caseload and service delivery were assessed using process evaluation logs. **Results**: Twenty-two professionals (17 GPs, two RPMWs, and three PCZ staff members) completed the NoMAD questionnaire. Intervention familiarity increased during the first eight months (T0–T1: *p* < 0.001; T1–T2: *p* = 0.022) and continued to rise thereafter (T3–T4: *p* = 0.008). Integration into daily practice and perceived impact on professional work improved progressively, reaching near-ceiling scores. Peer review sessions highlighted the RPMW’s central role in trust-building and care coordination. Over 12 months, one full-time equivalent RPMW supported 175 patients (mean age 40.7 years; 75% female), with an average of five consultations per patient. Patients reported high satisfaction, emphasizing accessibility, empathy, and practical support. **Conclusions**: Sustained results suggest successful normalization and support the potential of collaborative, low-threshold depression care. Future work will assess clinical and economic outcomes.

## 1. Introduction

Globally, depressive disorders affect over 322 million people [1]. Lifetime prevalence is estimated at 11–18% [2,3,4], with a higher prevalence observed in women compared to men [5]. The prevalence is increasing over time [6]. A first depressive episode frequently occurs during adolescence, with prevalence rates peaking in the second and third decades of life [4]. Depression is the leading cause of suicide and constitutes the single largest contributor to disease burden when measured in years lived with disability [7]. The disorder is characterized by substantial clinical complexity, partly due to substantial comorbidity with a wide range of health conditions [8].

Substantial barriers often hinder access to mental healthcare, resulting in delayed treatment initiation. Only 40% of adults with mental health disorders receive professional support within one year of symptom onset, with financial constraints and societal stigma frequently cited as primary obstacles. Although individuals with depression are somewhat more likely to seek help compared to those with other psychiatric disorders, a major impediment remains the belief that they can resolve their problems themselves [9,10]. Once care is sought, patients often have to wait weeks to months before receiving psychotherapeutic treatment. During these delays, there is a concern regarding symptom trajectories, with 42% of patients experiencing persistent symptoms and 56% experiencing worsening symptom severity [11,12].

While some individuals may experience spontaneous remission [13], not all patients have the opportunity to recover without treatment. It is challenging to make forecasts on who will recover and who will not. Moreover, depression becomes chronic with recurrent episodes in 33–66% of cases [14]. Given the significant mortality, morbidity, and unpredictable course associated with depressive disorders, sustained efforts to improve prevention, timely detection, and treatment remain imperative.

The increasing prevalence of depression is striking, given the decades of effort in improving pharmacological and psychotherapeutic interventions. A recent meta-analysis showed that depression outcomes have not improved over time [15]. One plausible explanation is that research and clinical practice have historically placed disproportionate emphasis on the specific components of psychotherapeutic interventions, despite these accounting for only an estimated 17% of the overall treatment effect. Common therapeutic factors, such as the quality of the therapeutic alliance, appear to drive treatment outcomes substantially more, alongside mechanisms such as spontaneous remission, regression to the mean, and contextual or environmental influences [13,16,17,18]. These observations suggest that further improvements in outcomes are unlikely to be achieved by doing more of the same. Instead, a paradigm shift might be warranted, emphasizing low-threshold, continuous, and coordinated care embedded within a robust primary care system [9]. Indeed, primary care settings have been determined as the preferred healthcare platform for the diagnosis and management of depression [19].

Therefore, the Belgian Integrated Depression Care (IDECA) project had been set up with the financial support of Johnson & Johnson. IDECA aimed to implement and evaluate an integrated care approach, rooted in primary care with linkages to the community level and more specialized care services. Rather than focusing on medical or therapeutic-technological novelties, IDECA’s innovation lies in optimizing and integrating existing care structures by strengthening general practice and enhancing collaboration across primary and secondary healthcare providers. IDECA adhered to the collaborative care definition with a multiprofessional approach to patient care, structured management plan, scheduled patient follow-ups, and enhanced interprofessional communication [20]. Such complex interventions have been found to improve mental health outcomes [21], although most research has been conducted in the United States and the United Kingdom. IDECA has been implemented in the Flemish region of Belgium.

A concise overview of Belgian mental healthcare epidemiology and existing services can be found in Willems et al. [22] Relevant to this paper is that depression prevalence in Belgium seems to be higher (9%) compared to international standards (5–8%) [23]. Some lingering effects of the COVID-19 period persist to today [24]. Over one in eight adults take antidepressants [25]. Unmet care is an issue, with half of the people with clinically assessed mental health needs not accessing healthcare services while actually requiring them [10].

Although Belgium is still known for having a high number of residential psychiatric beds, a shift towards more intensification and community care has taken place in the past decades. Alternative forms of care, such as mobile teams and the establishment of mental healthcare networks, have been developed [22]. In parallel, integrated community care has been strengthened in the last 10 years by the establishment of 60 primary care zones (PCZs) in Flanders and Brussels. A PCZ is a geographically defined network of primary care and welfare providers (e.g., GPs, social workers, psychologists), as well as patient representatives and local authorities delegations that coordinate their activities and exchange knowledge to meet local needs and improve the quality of care. Each zone covers at least 60,000 inhabitants and is governed by a care council [26].

A mixed-method approach, involving process evaluation, logbooks, focus groups, individual interviews, and questionnaires, has been implemented to evaluate IDECA’s feasibility, effectiveness, and affordability. This paper describes the co-creative development of the IDECA collaborative intervention and how it has been appreciated by both healthcare professionals and patients. Future papers will address clinical and economic outcomes.

## 2. Materials and Methods

A more extensive description, including scientific justification (if applicable) of setting, intervention development, components and materials, study design, and evaluation tools, can be found on Open Science Framework [27] or in Supplementary Materials.

### 2.1. Setting

IDECA has been implemented in the Flemish region of Belgium. Following a selection procedure, facilitated by the Flemish Institute for Primary Care (VIVEL), IDECA was implemented in two PCZs. PCZ Mechelen–Katelijne (PCZ MK) covers an area with 108,523 inhabitants and a higher-than-average population density. The population is younger than the average for the Flemish Region, with an average socio-economic status, and a higher uptake of general medical records; the zone is served by 79 general practitioners (GPs) in urban Mechelen and five practices in rural Sint-Katelijne-Waver [28].

PCZ Voorkempen (PCZ VK) encompasses 110,795 inhabitants with a lower overall density, but strong variation exists between municipalities. The PCZ faces more pronounced aging compared to the Flemish Region. Its socio-economic profile broadly compares to the regional average, with slightly higher employment rates, and the area is served by 91 active GPs and 18 GP trainees [29].

In PCZ MK, four general practices participated, ranging from a solo practice with part-time collaborations (e.g., nurse, psychologist, speech therapist) to larger multidisciplinary group practices. These included a practice with three GPs and nursing and psychological support, a practice with seven GPs (including two trainees) offering facilities for a wide range of other health professionals, and a practice with four GPs, a GP trainee, and two nurses. In PCZ VK, three practices contributed: a medical center with two GPs, a movement coach, a personal coach and a psychologist; a group practice of six GPs including two trainees, allied health professionals (physiotherapist, dietitian, children’s coach), and a psychotherapist; and a smaller center with one GP, one trainee, two psychologists, and a specialized nurse.

### 2.2. Intervention

**Figure 1:**
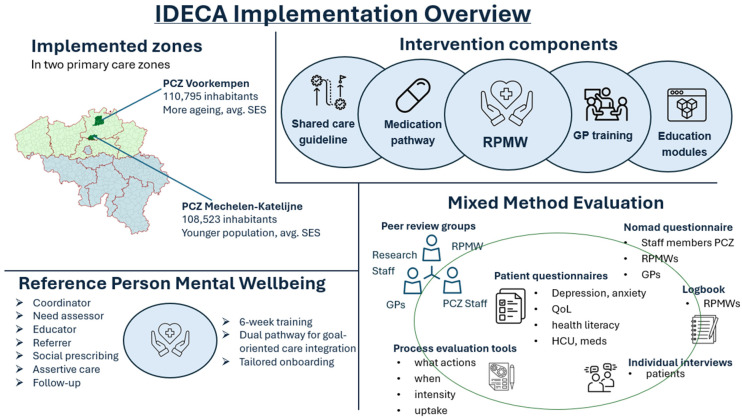
IDECA implementation overview. Developed by the authors in Microsoft PowerPoint.

IDECA was developed with five key components aimed at improving depression management in primary care. A key element was the integration of a Reference Person Mental Wellbeing (RPMW) into general practices, acting as a case manager to support patients and GPs. Additional elements included tailored self-management education modules, a shared care guideline to enhance coordination across care levels, a medication pathway for evidence-based pharmacological decisions, and GP training to promote integrated, person-centered care (Figure 1).

***RPMW (case management).*** One full-time equivalent RPMW was implemented over 18 months (one half-time per PCZ), collaborating with selected GP practices. One RPMW had an occupational therapy background, and one was a psychologist by education. The RPMWs acted as mental health generalists, working within GP practices while linking them to community and specialized services. They complemented existing primary care facilities by supporting the assessment and diagnosis of patients’ needs, by referring to psychosocial and lifestyle services, by arranging long-term follow-up, and by providing assertive care. Patients were supported through flexible follow-up appointments, either in person or remote. The RPMW also provided goal-oriented support to patients, encouraged social prescribing, and facilitated medication adherence by addressing barriers and relaying insights to GPs. Prior to patient inclusion, RPMWs completed a six-week training program covering the IDECA framework, integrated care, monitoring tools (e.g., OQ-45), depression education modules, recovery-oriented communication, and use of the Zipster social prescribing tool (in PCZ MK only) [30], mapping of local care resources, and goal-oriented care (GOC). GOC is an approach that aligns care with what matters most to patients by focusing on their personal goals and preferences [31]. Accordingly, the GOC training program for RPMWs aimed to build competencies to foster meaningful and coordinated care, thereby contributing to person-centered integrated care. Two complementary pathways were implemented: an individual RPMW-centered pathway comprising three skill-building sessions, and a PCZ-centered pathway comprising three peer review sessions guided by Normalization Process Theory [32]. Together, these pathways fostered RPMW skill development, role delineation, and integration of GOC within local primary care teams. The onboarding process was tailored to each RPMW’s background and included continuous peer review. For example, the RPMW with an occupational therapy background received more education on depression, while the RPMW with a psychology background received more focus on mapping social resources.

***Self-management education.*** A patient education map [33], originally developed by a collaboration of patient organizations, healthcare professionals, and Johnson & Johnson (Beerse, Belgium), was redesigned to six educational modules (i.e., “What is depression?”, “Treatment & support”, “For relatives”, “Suicide”, “Talking to your children”, and “Life after depression”). These resources aimed to increase patients’ health literacy and empowerment, and enhance shared decision-making.

***Shared care guideline.*** A professional decision-tree-based tool was created to visualize and supplement the existing depression care recommendations from the family medicine organization Domus Medica [34]. The tool embedded the RPMW role and outlined diagnostic, therapeutic, and referral pathways across different care levels. Validated by a scientific steering committee, it was distributed in print and online to support collaborative care while remaining non-prescriptive.

***Medication pathway.*** A visualized tool for antidepressant prescribing was developed, validated by two psychiatrists, and made available in print and digital formats. It supported GPs in considering pharmacological options, monitoring responses, and adapting treatment while acknowledging individual patient variability.

***GP training.*** Two accredited refresher sessions were organized and delivered by experts in psychiatry and integrated care. The sessions covered depression management, population approaches, integrated shared care, and medication management.

### 2.3. Study Design and Patient Population

IDECA adopted a single-arm implementation design, primarily focusing on optimization, feasibility, and acceptability in a real-world setting. Patients were broadly defined as adults diagnosed with depression by their GP in general practice, based on a priori defined but non-restrictive guidelines and the GP’s own clinical evaluation. Few exclusion criteria applied, except for age (under 18 or over 65) and specific conditions requiring specialized care (e.g., psychosis, bipolar disorder, high suicide risk). Characteristics of enrolled patients are presented in Section 3.2.2. The aim was to ensure ecological validity by defining the patient population bottom-up along the road, based on GPs’ experience and assessment. Moreover, the above-described new RPMW role was incepted as a guiding framework. Throughout the intervention, our goal was to monitor and co-create the naturally evolving role, based on participants’ experience and context-specific needs.

### 2.4. Data and Analysis

IDECA comprised a mixed-method approach combining qualitative and quantitative approaches. The current paper aims to assess the co-creation process and stakeholder appreciation of the intervention and its feasibility. Only part of all the data collected was considered relevant to answer the current paper’s research question and was thus taken into account.

To map the implementation process from the perspective of the healthcare providers, a modified version of the NoMAD questionnaire was used [35], which can be consulted on OSF [27]. This instrument was developed to assess how new technologies and complex interventions are adopted and integrated into healthcare settings. Part A collects respondent background information. Part B contains general questions on innovation normalization and process factors. Part C focuses on the four core constructs of Normalization Process Theory (NPT): coherence, cognitive participation, collective action, and reflexive monitoring. The NoMAD was administered every four months: at baseline (T0, two weeks after start of the intervention), and subsequently at 4 (T1), 8 (T2), 12 (T3), and 16 (T4) months. Responses were recorded on Likert scales. Answers to Part B were treated as numerical data; visual inspection of QQ-plots and histograms of paired differences indicated non-normality, hence the Wilcoxon signed-rank test was used for statistical inference. Responses to Part C were ordinal in nature, for which the Wilcoxon signed-rank test was also applied. Statistical significance was set at *p* < 0.05. Analyses were performed using SPSS Statistics version 29.

Additionally, the first two peer review sessions in each PCZ (90–120′) involving RPMWs, GPs, and PCZ staff were analyzed; the first one was organized 2–4 months after the first patient inclusion, and the second one after 6.5–7.5 months. The peer review sessions aimed to change immediate practice, congruent with the interpretative description method [36]. The first session aimed to map potential care trajectories for patients supported by the RPMW and to identify the conditions required for optimal implementation. The discussion guide was structured into four themes: (i) profile of patients who should be referred to the RPMW and why; (ii) role distribution within the support process; (iii) patient follow-up throughout the trajectory; and (iv) participants’ views on how the project might influence routine practice. The second session focused on the active integration of the RPMW into primary care. Its discussion guide included two main themes: (i) the course of the patient trajectory, emphasizing the balance between connecting care and the process of letting go; and (ii) collaboration and referral between the RPMW, GPs, and other professionals within the broader care network. A member check took place at a third peer review session, and feedback was sought on a written report. Some baseline characteristics from the process evaluation data further specify the patient profile.

To explore patient experiences, semi-structured interviews were conducted with individuals who had received support from the RPMW. On 20 September 2024, RPMWs each recommended ten patients who were subsequently invited by email, and on 13 November, an additional fifteen patients were invited at random. In total, eight patients participated, corresponding to a response rate of 23% (8/35 invited; 201 patients had been seen overall). Interviews were scheduled in a phased manner, allowing interim evaluation of thematic saturation. The semi-structured interview guide covered four main themes: (i) the course of depressive symptoms, (ii) expectations and experiences regarding the RPMW, (iii) psychoeducation, and (iv) perceived gaps in the healthcare system. Interviews were conducted by either R.W. or L.B., lasted 30–60 min, and were recorded and transcribed verbatim. The interviewer probed for concrete examples to ensure adequate context and interpretation. Data were analyzed using phenomenological thematic analysis as described by Braun and Clarke [37]: (1) data familiarization through transcription, (2) open coding of raw data, (3) axial coding to major data-driven inductive themes, (4) review of themes, and (5) interpretation of themes. Author R.W. conducted the first analysis (steps 1–3), after which author L.B. reviewed candidate themes and interpreted the thematic tree while rereading transcripts and coded extracts (steps 4–5). The process was iterative, with feedback loops between R.W. and L.B., to resolve any existing discrepancies. This approach emphasizes participants’ lived experiences, as captured through their subjective accounts [38]. The coding process was inductive, resulting in a thematic tree with main themes and subthemes. A member check with the patients did not take place.

### 2.5. Independence Statement

This study was funded by Johnson & Johnson. The investigators and funder collaborated throughout the whole study period, in which the funder had an advisory role. However, an independent scientific advisory committee was established as well to discuss intervention components. Final decisions on the intervention content and data analysis were the sole responsibility of the core investigator team (R.W., K.V.d.B., L.B.).

## 3. Results

### 3.1. NoMAD: Normalization Process

**Figure 2:**
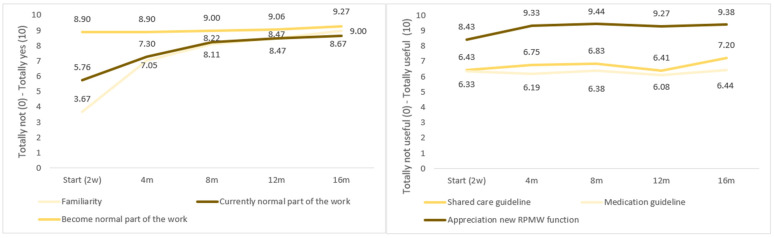
Innovation normalization over time (**left**); process factors over time (**right**).

Twenty-two respondents (17 GPs, 2 RPMW, and 3 staff members) filled out the NoMAD questionnaire (N on T0-1-2-3-4: 21-20-18-17-15, respectively). Figure 2 and Table 1 show that the intervention seemed practically feasible as familiarity with the intervention increased significantly in the first eight months (T0–1: +3.88, *p* < 0.001; T1–2: +1.06, *p* = 0.022) and continued to increase even in the final four months (T3–4: +0.53, *p* = 0.008). From the beginning, people felt that it could be embedded in routine practice, and in the first eight months, the intervention became a normal part of work (T0–1: +2.54, *p* < 0.001; T1–2: +0.98, *p* = 0.047).

GPs and staff members appreciated the RPMW function from the start (8.43), and a subsequent significant increase was noted in the first four months (+0.90, *p* = 0.027) after which a plateau was reached. Appreciation of the shared care guidance increased slightly but insignificantly between T0 and T4 (+0.77 to 7.20, *p* = 0.447). Appreciation of the medication guideline was consistently moderate over time (6.08 to 6.44).

**Figure 3:**
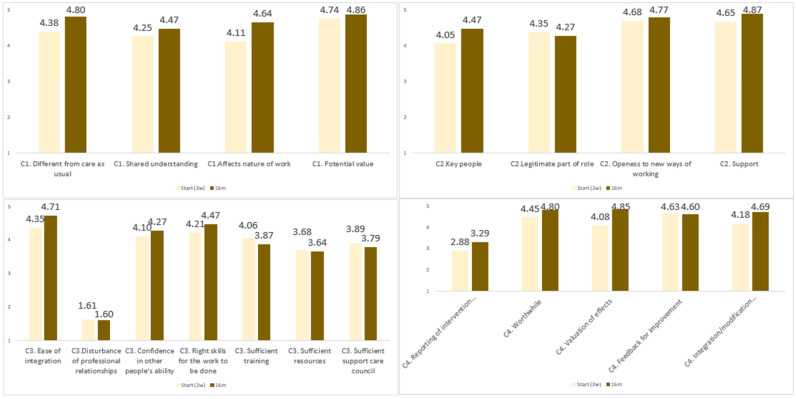
Four core constructs of Normalization Process Theory (NPT) at start (T0) and at 16 months (T4): coherence (**upper left**), cognitive participation (**upper right**), collective action (**bottom left**), and reflexive monitoring (**bottom right**).

Few significant effects were found on the four NPT core constructs: coherence, cognitive participation, collective action, and reflexive monitoring. Nevertheless, there was a clear positive trend in the appreciation of IDECA (Figure 3 and Table 1). The components showing statistically significant changes point to meaningful effects, particularly considering the limited statistical power due to the small sample size and ceiling effects on most measures.

Notably, there was a significant increase in the perceived value of the intervention on the work performed, which was already high at the start (4.08) and grew to a near-perfect score of 4.85 (*p* = 0.031). This increased valuation was achieved in the first four months of the intervention (+0.50, *p* = 0.047) and continued to increase over time. This may be linked to the ease with which the intervention can be implemented, given that it is modifiable to current practice.

The intervention was delivered by competent professionals, yet further scale-up will require additional attention to training opportunities, adequate resources, and strong support from the board of the PCZ (although these aspects were also evaluated positively).

### 3.2. Peer Review Groups and Process Evaluation

Each peer review group was attended by 2–4 scientific staff members, the RPMW, a PCZ staff member, and 3–4 GPs.

#### 3.2.1. Profile of the RPMW

Participants consistently emphasized that the RPMW constitutes a unique and autonomous professional profile within GP practices, characterized by being a trained generalist with an integrated care approach, and under the medical responsibility of the GP. For patients requiring psychotherapeutic treatment, referral to a clinical psychologist remains appropriate. Yet, compared with psychologists, the RPMW operates more broadly across different life domains, assessing severity and context. As one RPMW explained:

*“The metaphor is snorkeling versus deep-sea diving. The RPMW is snorkeling, a psychologist dives deep.”*

*(RPMW)*

Although individual RPMWs may differ depending on their backgrounds, consensus emerged on the minimal requirements. Entry requirements include several years of professional experience (in mental healthcare), as well as a paramedical or experiential background. Core competencies include knowledge of psychological processes and conditions (e.g., trauma, mood disorders, developmental problems), empathic communication skills, familiarity with integrated care and local welfare resources, didactic and follow-up skills, strong communication with other providers, and the ability to proactively coordinate continuity of care in a solution-oriented manner.

*“You have to interrupt sometimes and say: I’m not your psychologist nor just a listening ear. We’ll search specifically for solutions.”*

*(GP, PCZ MK)*

As the RPMW is defined as a function rather than a profession, people with different professional backgrounds may enter the role. This requires tailored training pathways. If the RPMW’s background is not primarily in mental health, greater standardization through structured tools may be required.

#### 3.2.2. Patient Profile

Process evaluation data showed that a twelve-month patient load per full-time equivalent accumulated to 175 patients. These patients were referred by GPs and had at least one contact with an RPMW, but demographic data were obtained of 57% of the sample (note that patient referral from the GP to the RPMW was ongoing, but that only the patients referred in the first eight months were invited for scientific follow-up). Average age was 40.7 years (SD 12.8; median 38, IQR 31–50), 75% identified as female and 24% as male, and 8% were born outside Belgium. More than half (56%) were married or cohabiting, and 39% held at least a higher education degree. Baseline PHQ-9 scores showed that 19% scored lower than 10 (i.e., mild depressive symptoms), while 28% scored 10–14 (i.e., moderate depression), 31% scored 15–19 (i.e., moderately severe depression), and 22% scored 20–27 (i.e., severe depression).

GPs did not refer patients with very low complexity, such as cases of simple exhaustion.

*“It should never become a program where everyone is advised to see the GP just to check if they feel okay and then get sent to the RPMW.”*

*(GP, PCZ VK)*

At the same time, GPs avoided referring highly complex psychiatric cases, recognizing that the RPMW’s input would not be efficient for patients with severe personality disorders or long-standing unsuccessful experiences in tertiary care.

*“Some patients come in with more than depression… with a personality disorder, you know you’ll be stuck for hours, and the RPMW will put in endless energy with zero result.”*

*(GP, PCZ MK)*

Referral was therefore focused on motivated patients, who were often financially vulnerable and with primary mood disorders, for whom GPs felt stuck. These patients did not necessarily require residential care but rather had previously experienced unsuccessful first-line treatment. RPMWs themselves noted that underlying traumatic or attachment-related experiences frequently surfaced during intake consultations:

*“You often get depressive complaints, but it is really about the traumas and the context underneath.”*

*(RPMW)*

#### 3.2.3. Tasks of the RPMW

The intake consultation aimed to explore different life domains to identify both dysfunctional and protective factors. These conversations established a trusting therapeutic relationship, enabling psychoeducation, motivational support, and tailored referral across the continuum of care.

*“I always ask: what gives you energy, or what used to give you energy? That way I can see what might be a good match for referral.”*

*(RPMW)*

Patients were encouraged to take responsibility for contacting other services themselves. However, the RPMW provided intensive guidance where needed, sometimes even accompanying patients to welfare or healthcare institutions in person. Ideally, the RPMW remained involved after referral as a companion and care coordinator.

*“That’s often mentioned as a point of appreciation: when referrals are made, the RPMW follows up and sometimes even accompanies the steps. It’s fantastic for patients to have someone they trust when taking that step.”*

*(GP, PCZ VK)*

*“The case management role of the RPMW is highly valued. Getting people started, following up, orienting, searching together… it takes time. And the RPMW does it much better than I ever could as a GP.”*

*(GP, PCZ VK)*

In summary, the RPMW role encompasses multiple interconnected tasks, including direct patient consultation, assessment of problem areas, psychoeducation, motivational and limited therapeutic support (depending on background competencies), referral to health and welfare services, and proactive coordination and follow-up.

Process evaluation data provides a quantitative picture of the RPMWs’ workload. Over 12 months, 879 contacts were made with 175 patients (an average of five contacts per patient), with an average consultation length of 57 min. Of these, 754 were in-person consultations of ≥20 min, lasting on average 65 min. The remaining contacts included phone calls, cancelations, and attempts to reschedule.

Some differences between settings could be observed. In PCZ VK, the RPMW saw 30% more patients than in PCZ MK (99 vs. 76), resulting in 36% more total contacts (507 vs. 372). However, consultations in PCZ VK were shorter on average (51 vs. 65 min), leading to only a 6% higher total consultation time across the year. Direct patient contact accounted for 50% of total working time (48% in PCZ MK and 51% in PCZ VK).

### 3.3. Patient Interviews

Table 2 shows the characteristics of the interviewed patients. Respondents were substantially older than the average patient population, and they had double the average number of consultations.

Patients most often cited depressive symptoms, burnout, and multiple stressors as reasons for referral to the RPMW, with loneliness and low self-esteem also commonly mentioned. Many had previously sought help from formal services, self-help programs, or alternative care, often without sustained benefit.

Patients were unanimously positive about the RPMW role, describing it as accessible, supportive, and impactful. Compared with psychologists, the RPMW was perceived as being more approachable, embedded in the community, and focused on practical solutions. A few recommendations were mentioned, such as the importance of knowledge of breathing technique and the more timely use of psychoeducation material. The presence in GP practices and the free-of-charge service lowered barriers to access and fostered trust.

*“And the fact that it was in the GP’s practice was an advantage. That made me trust it. It was easily accessible for me. It is clean and tidy. A real advantage.”*

*Respondent 1 (MK)*

Patients emphasized the RPMW’s conversational skills, such as listening, empathy, paraphrasing, and offering reassurance, as these facilitated trust and personal insight. Equally valued was a “hands-on” mentality, involving concrete advice, encouragement, and referrals to health, welfare, or community resources. This pragmatic approach helped patients reduce acute stress, re-engage in meaningful activities, and build connections.

*“Making suggestions was her strength. She asked me what I liked to do. I said swimming, and she encouraged me. And I actually started doing it. … She asked me what I wanted to do, and sometimes I said things I had not even thought of before.”*

*Respondent 6*

## 4. Discussion

The findings suggest that the IDECA collaborative model successfully bridged the gap between primary and mental healthcare. This was achieved by introducing a feasible and valued collaborative practice that became embedded in routine practice within one year. IDECA appeared to be both feasible and acceptable, showing immediate and progressive normalization over time. Familiarity with the intervention and its integration into routine practice increased significantly over time, suggesting that professionals quickly embraced and maintained its use. Despite limited statistical power and ceiling effects, these trends can be considered meaningful and consistent with the qualitative findings.

Collaborative practices are found to increase the chances for a depression response by about 30% compared to usual care [39]. Despite the potential, the gap between efficacy and implementation has to be taken in mind [40]. Overbeck et al. (2016) [41] identified several enablers and barriers for implementation. Buy-in of primary care providers and GPs in particular is key [41]. For instance, a fast rise in GPs’ and PCZ staff’s valuation of the RPMW role was noted, and this appreciation remained stable afterwards, reflecting the perceived added value of an accessible, generalist, and collaborative mental health function embedded in general practice. The great integration may be explained by the fact that IDECA incorporated several elements of practice-based care management, which have demonstrated better normalization compared to centralized care management [42]. Centralized care management is characterized by case managers working more independently and outside the practice. In practice-based care management, the GP refers the patient to the case manager, who is viewed as a competent and trustworthy in-house extension of the GP’s practice. IDECA built upon this component by introducing a six-week training period to strengthen the social and professional skills of the care managers; these skills are reported as critical for successful implementation [41]. These findings were confirmed by complementary qualitative data from our peer review groups and patient interviews: the RPMW was described as a unique, hands-on, and trust-building role bridging medical, psychological, and social care. This aligns with the perspective in which recovery includes clinical, functional, social, and personal domains [43].

Patients and professionals alike reported improved continuity and coordination. The appreciation of patients is in line with the reported increased patient satisfaction rates following collaborative care practices [39], and may have resulted in improving patient adherence by overcoming structural barriers [44]. The co-location of the RPMW within GP practices was another enabler, as suggested before by Overbeck et al. [41], fostering trust and face-to-face interactions between caregivers. This is of key importance because integrating a new professional function may entail certain risks, including task shifting, role ambiguity, and a higher organizational complexity.

The RPMW has been conceptualized as a function rather than a fixed profession, which allows flexibility but necessitates careful attention to role clarity. While individual RPMWs may have diverse professional backgrounds, consensus was reached on minimal entry requirements and a defined set of core competencies, including experience in mental healthcare, knowledge of common psychological conditions, strong communication and coordination skills, and familiarity with integrated care and local welfare resources. This aligns with comparable roles such as the Dutch POH-ggz, where professionals from varied educational backgrounds operate within primary care [45].

IDECA developed a guiding framework for GPs for patient inclusion, but the actual patient population needed to be defined bottom-up based on the professional judgment of GPs. Especially senior workers may appreciate such clinical freedom. A strict depression definition can be too narrow to apply in practice, given that co-morbidity is prevalent [40]. Indeed, the RPMWs often detected underlying traumatic or attachment-related experiences in referred patients. Moreover, a co-existing chronic physical condition should not hamper GPs from referring people with depression to the RPMW, as collaborative care is found to be equally effective in populations having a co-existing chronic physical condition or not [46]. This is of particular interest as IDECA’s guiding framework excluded patients above 65, an age group well-known for co-morbidities. Shulman et al. [47] demonstrated the feasibility and the need to extend such collaborative practices to the elderly with depression, possibly at a reasonable value for money [48].

Moreover, 8% of the patients in IDECA were born in a country other than Belgium. Although not registered, it is not unthinkable that others have a migrant background too, since one in six people in Flanders has [49]. Especially, people with a Moroccan or Turkish background in Belgium face significantly more mental health disparities compared to non-migrant Belgians [50]. No specific cultural adaptations were implemented within the IDECA, but specific training on cultural reflexivity, the possible pivotal place of spirituality and religion, and the possible additional psychological burden in these patients might be advisable to further strengthen integration of care [51].

IDECA might thus be valuable for a broad range of patients, underlining its potential scalability. Equally important for scalability is the budget impact of a new intervention, which will be addressed for IDECA in a future publication. An intervention may be seen by both patients and professionals as feasible and valuable, which are necessary prerequisites, but should be supplemented by a thorough analysis of patient-reported outcomes and costs. Klaehn et al. (2022) [52] found, for instance, that case management in a range of diseases has shown overall promising but varying health economic impact.

Although developed within a high-income country, IDECA’s collaborative and task-sharing principles align with the WHO’s mhGAP framework on mental healthcare in primary care through structured training, stepped care, and non-specialist delivery models [53]. These principles might also be applicable in low-to-middle-income countries or even regions in crisis where specialized services can be scarce. Trained and supervised non-specialist providers (such as lay or community health workers) might effectively deliver depression care [54,55].

### Study Limitations

This study has several limitations. IDECA was developed with strong ecological validity but limited internal validity. The single-arm design precludes causal conclusions, as the primary focus was normalization rather than clinical effectiveness. Implementation in two PCZ with a limited number of professionals may restrict generalizability and may reflect local motivation and contextual support. Interviewed patients may not represent the broader population. Missing data occurred at both provider and patient levels due to the pragmatic embedding of research procedures, and some aspects of the NoMAD questionnaire require further evaluation. We elaborate upon these limitations in the following paragraphs.

First, IDECA’s intrinsic value undoubtedly lies in the strong contemporary scientific foundation of its components and how these were integrated. Ecological validity was strived for, as evidenced by the co-creative design of the RPMW’s role, and the step-by-step construction of the patient profile based on GPs’ experience and assessment. Indeed, the intervention appeared adaptable to existing workflows, as indicated by significant gains in perceived ease of integration and the valuation of its impact on daily work. However, the downside of this approach may be a limited internal validity.

Second, a single-arm design was chosen, as demonstrating effectiveness was not the primary aim of the IDECA study. The absence of a comparator limits the possibility of drawing causal inferences regarding the added clinical value of the intervention and restricts the interpretation of its broader relevance. In any case, the use of control groups in conventional randomized trials may have come to a concluding point in psychosocial interventions, as some have argued recently. There is no good use of control conditions because waitlist controls may overestimate effects, generalizability is limited by heterogeneous care as usual practices, and placebo use raises pertinent questions, including ethical ones [56]. Nevertheless, when outcome data on, for instance, depression, quality of life, health literacy, medication adherence, and healthcare utilization will be reported in separate papers, the lack of a comparative design will limit inferences on relative benefits.

Third, IDECA has only been implemented on a modest scale in two regions. This equates to two RPMWs, together one full-time equivalent, supporting seven GP practices. Arguably, selection biases on both the PCZ and healthcare professional levels are present, possibly limiting generalizability. The RPMWs and GPs were supported by the PCZ staff, which was a selection criterion to participate in the IDECA project. This suggests that contextual readiness, local leadership, and intrinsic motivation may have contributed substantially to successful integration. It is therefore important to note that it is not only the intervention components that make the intervention work, but it is also the people cooperating and choosing to make it work (paraphrase on Pawson and Tilley, 1997 [57]). Generalizability on the patient level may also be limited. Compared with epidemiological data from Flemish primary care, our sample included a higher proportion of women (75% vs. 62–65%) and a slightly younger population (40.7 vs. 44.3–45.2 years) [58]. Although both studies reflect the higher prevalence of depression among women, the overrepresentation of female and somewhat younger patients suggests potential selection bias.

Fourth, interviewed patients may not be representative of the whole patient population. Indeed, patients willing to participate in the interviews have had more contact time with the RPMW than on average. Inclusion of negative deviant cases, such as patients with only one visit or young adults, might have enriched the data. Saturation was now already achieved after six interviews, after which two more were conducted, which approaches the number of required interviews (9) for theme saturation as recommended by Wutich et al. [59].

Fifth, dropout occurred at both the healthcare provider and patient levels. Among providers, some GPs did not complete all questionnaires despite remaining engaged with the intervention. At the patient level, substantial missing data were evident, including at baseline. This was partly due to patients not returning questionnaires to the RPMW, and partly because RPMWs, whose primary focus was on delivering care, occasionally overlooked the scientific data collection procedures. Quantitative analyses in this paper were therefore interpreted descriptively. Future papers on clinical patterns will account for missing data by multiple imputation techniques.

Sixth, the NoMAD questionnaire received a fair global validity score with excellent content validity and fair structural validity. However, hypothesis testing, cross-cultural validity, and criterion validity have yet to be assessed [60].

## 5. Conclusions

Both patients and healthcare workers valued IDECA. The growing appreciation indicated a shift toward more integrated ways of working, with a tangible impact on daily operations. Several key figures fostered strong engagement with the intervention. However, this study focused on implementation processes rather than clinical indicators. While normalization is a necessary precondition for impact, it does not in itself demonstrate improved patient outcomes. Upcoming studies will provide a broader picture by focusing on clinical and economic outcomes, as well as process evaluation measures. This is necessary to determine whether integration, appreciation, and engagement of the intervention result in measurable patient-reported outcomes.

## Figures and Tables

**Table 1:** NoMAD Wilcoxon exact tests (*p*-value ≤ 0.05).

	T0–1	T1–2	T2–3	T3–4	T0–4
Familiarity *How familiar does IDECA feel?*	**<0.001**	**0.022**	0.973	**0.008**	**<0.001**
Currently a normal part of work *Does IDECA feel like a normal part of your work?*	**<0.0001**	**0.047**	0.67	0.408	**<0.001**
Become a normal part of work *Do you think IDECA could become a normal part of your work?*	0.793	0.375	0.938	0.359	0.234
Shared care guideline *How helpful do you find the shared care guideline?*	0.379	0.986	0.516	**0.047**	0.447
Medication guideline *How helpful do you find the medication pathway?*	0.811	0.453	0.438	0.906	0.961
RPMW *How helpful do you find the RPMW?*	**0.027**	0.375	0.828	0.5	0.281
C1. Different from care as usual *I can see how IDECA differs from the usual way of working.*	1	0.289	1	0.25	0.125
C1. Shared understanding *Employees within my organization have a shared understanding of the purpose of IDECA.*	1	1	0.75	0.375	0.125
C1. Affects the nature of work *I understand how IDECA affects the nature of my work.*	0.234	0.289	1	0.688	0.188
C1. Potential value *I see the potential value of IDECA for my work.*	1	0.5	1	1	1
C2. Key people *There are key people who drive IDECA forward and engage others.*	0.781	1	1	0.688	0.188
C2. Legitimate part of the role *I believe that participating in IDECA is a legitimate part of my professional role.*	0.754	0.07	0.688	1	0.809
C2. Openness to new ways of working *I am open to working with colleagues in a new way in order to make use of IDECA.*	0.75	0.5	0.375	0.5	1
C2. Support *I will continue to support IDECA.*	0.375	0.625	0.625	1	0.375
C3. Ease of integration *I can easily integrate IDECA into my existing workload.*	1	**0.039**	1	1	0.125
C3. Disruption of professional relationships *IDECA disrupts existing professional relationships.*	1	1	1	1	1
C3. Confidence in other people’s ability *I have confidence in the skills of others to implement IDECA.*	1	0.07	1	0.125	1
C3. The right skills for the work to be done *The work is allocated to people with the right skills to deliver care.*	0.125	1	1	1	0.75
C3. Sufficient training *Sufficient training is available to implement IDECA.*	1	0.75	0.25	1	0.125
C3. Sufficient resources *Sufficient resources are available to implement IDECA.*	0.359	0.75	1	0.75	1
C3. Sufficient support care council *The care council supports IDECA in an adequate way.*	0.375	0.25	0.727	1	0.594
C4. Reporting of intervention results *I am aware of reports on the effects of IDECA.*	0.398	0.828	0.75	0.234	0.48
C4. Worthwhile *IDECA is valuable.*	0.375	0.375	1	1	0.18
C4. Valuation of effects *I value the effects IDECA has had on my work.*	**0.047**	0.688	1	1	**0.031**
C4. Feedback for improvement *Feedback on IDECA can be used to further improve the intervention in the future.*	1	0.625	1	1	1
C4. Integration/modification possibility in practice *I can adapt the way I work to the IDECA elements.*	0.563	0.125	1	1	0.063

Bold indicates significance on the 0.05 level. Italic sentences are translations of the NoMAD statements.

**Table 2:** Characteristics of interviewed patients.

ID	Gender	Age	PCZ	Contact Time (Days)	Number of Contacts
1	F	58	MK	511	21
2	F	56	VK	245	15
3	F	60	MK	175	7
4	F	60	MK	497	18
5	M	44	MK	287	12
6	F	44	VK	189	9
7	F	61	VK	433	14
8	F	61	VK	245	11
Average		55.5		322.8	13.4
SD		6.8		128.0	4.3

## Data Availability

Data is not open-access due to privacy restrictions, but can be obtained from the authors on reasonable request. The Open Science Framework project p9mrq was made publicly available on 10 November 2025 [27].

## Supplementary Materials

The following supporting information can be downloaded at: https://www.mdpi.com/article/10.3390/jcm15062326/s1, (Willems et al. supplementary material_elaborated intervention description), Figure S1. Information guide developed by Ups & Downs in collaboration with Janssen-Cilag. Left: cover. Right: extract. Figure S2. Shared care guideline (in Dutch). A PDF version in better resolution is accessible on OSF. Figure S3. Medication path (in Dutch). A PDF version in better resolution is accessible on OSF. Figure S4. Major depressive disorder specifiers. Figure S5. Intervention area recruitment process. Figure S6. Overview mixed-method evaluation tools. Table S1. Training Content and Action Plan for RPMW Onboarding. Table S2. Attendees at peer review sessions. Table S3. Semi-structured interview guide, freely translated from Dutch to English. References [4,27,28,29,33,34,35,37,38,61,62,63,64,65,66,67,68,69,70,71,72,73,74,75,76,77,78,79,80,81,82,83,84,85,86,87,88,89,90,91,92] are cited in the supplementary materials.

## Abbreviations

The following abbreviations are used in this manuscript:

PCZ	Primary Care Zone
RPMW	Reference Person Mental Wellbeing
IDECA	Integrated Depression Care
NPT	Normalization Process Theory
MK	Mechelen–Katelijne
VK	Voorkempen
OSF	Open Science Framework
