# Supplementary material for: Development, Feasibility, and Appreciation of the Collaborative Integrated Depression Care (IDECA) Project in Flanders, Belgium
Source: J Clin Med. 2026 Mar 18;15(6):2326. doi: 10.3390/jcm15062326 (PMC13027223; doi:10.3390/jcm15062326)
Supplement: Supplementary file 1 [file jcm-15-02326-s001.zip › jcm-4162402-supplementary.pdf]

Supplementary material

elaborated intervention description

Ruben Willems [a] [*], Kris Van den Broeck [b], Reini Haverals [c], Lieven Annemans [a], Pauline Boeckxstaens [c], Didier Schrijvers [d e], Geert Goderis [f], & Liesbeth Borgermans [a]

[a] Interuniversity Center for Health Economic Research (I-CHER), Unit of Health Economics and Management, Department of Public Health and Primary Care, Ghent University, Ghent, Belgium

[b] Department of Family Medicine and Population Health, University of Antwerp, Antwerp, Belgium

[c] Unit of Family Medicine, Department of Public Health and Primary Care, Ghent University, Ghent, Belgium

[d] Department of Psychiatry, Collaborative Antwerp Psychiatric Research Institute (CAPRI) and Antwerp University Hospital, Faculty of Medicine and Health Sciences, University of Antwerp, Antwerp, Belgium

[e] Department of Psychiatry, University Psychiatric Center Duffel, Duffel, Belgium

[f] Department of Public Health and Primary Care, KU Leuven, Leuven, Belgium

Version and date: 1.0, August 5th 2025

Table of contents

TABLE OF CONTENTS **2**

AIM **3**

INTERVENTION COMPONENTS **3**

1. REFERENCE PERSON MENTAL WELLBEING (CASE MANAGEMENT FUNCTION) 3
   - *The RPMW as coordinator* *4*
   - *The RPMW as supporter and motivator* *5*
   - *The RPMW as information provider: self-management education modules* *5*
   - *Training of RPMWs* *6*
2. SHARED CARE GUIDELINE 13
3. MEDICATION PATHWAY 16
4. GP TRAINING 18

RESEARCH DESIGN **18**

SINGLE ARM IMPLEMENTATION 18
HETEROGENEOUS PATIENT POPULATION CALLS FOR A REAL-WORLD APPROACH 19
INTERVENTION AREA RECRUITMENT 20
INTERVENTION AREAS 21
PARTICIPATING GENERAL PRACTICES 22
SELECTION OF THE RPMW 22

EVALUATION TOOLS **23**

QUANTITATIVE: PATIENT QUESTIONNAIRES 23
QUANTITATIVE: NOMAD 24
QUALITATIVE: FOCUS (PEER REVIEW) GROUPS 24
QUALITATIVE: INDIVIDUAL INTERVIEWS 25
PROCESS EVALUATION 27
LOGBOOK 27

REFERENCES **28**

Aim

The integrated depression care (IDECA) project aimed to scrutinize the potential feasibility of a real-world implementation of the IDECA intervention. The intervention consisted of different intervention components with the integration of a case management function in general practices as central pillar. The following research questions are aimed to be answered:

- Which components of the IDECA-intervention are supported by healthcare providers? How does this evolve over time?
- What are the patient characteristics of patients with depressive symptoms that are referred by the general practitioner to the Reference Person Mental Wellbeing (RPMW; the case manager)?
- What is the function classification of the RPMW? How does the role evolve over time and in co-creation?
- Do patients perceive the IDECA intervention components as helpful, and which patients do or do not experience it as such?
- What contextual conditions must be met within a given setting for the IDECA intervention to provide added value?
- What is the actual dose intensity of the intervention in practice (how many patients can be helped by one full-time equivalent (FTE) RPMW in a reference period)?
- What is the budget impact of implementing the IDECA intervention?
- What is the association between the IDECA-intervention and clinical pathways of included patients?

Intervention components

The IDECA intervention has been built around five core components designed to strengthen the management of depression in primary care. Central to the approach was the integration of an RPMW within general practices, acting as a case manager to support both patients and healthcare providers. The intervention also included self-management education modules tailored for patients and their close contacts, promoting empowerment and a better understanding of depression. To encourage collaborative care across different levels of the health system, a shared care guideline was developed and implemented, providing practical guidance for care coordination. In addition, a medication pathway was created to support evidence-based pharmacological decision-making, primarily for general practitioners. Finally, dedicated training for GPs ensures that all healthcare providers involved are equipped with the knowledge and tools needed to deliver integrated, person-centered care.

1.Reference person mental wellbeing (case management function)

The first and most crucial pillar of the IDECA intervention is the integration of a case management function into the healthcare landscape to support general practitioners and promote interprofessional collaboration. The purpose of case management was to facilitate the care process in such a way that patients receive the necessary care and support[1]. It was essential that this function was embedded in the network in a complementary manner.

IDECA has implemented one FTE RPMW for 18 months, thus a half time FTE in each intervention area. The original aim was to collaborate with 2-4 general practices (+/- 10 GPs) per intervention area.

The case management role can take on various forms depending on the nature of the problems addressed and the care setting. Much of the scientific evidence supporting this role originates from Anglo-Saxon countries[2,3].

Within the IDECA project, the case manager, referred to as the RPMW, acted as both advisor and mediator[1]. At the start of the project, a broad and integrated set of responsibilities was envisioned for this role, based on literature, international expertise consultation, and a scientific steering committee including general practitioners, a psychiatrist, psychologists, nurses, an expert by experience, and a health economist.

Note that the specification of the RPMW role was subject to investigation. Via co-creating intervention sessions with the RMPWs, PCZ staff members en GPs, the role was further defined throughout the intervention.

The RPMW as coordinator

as coordinator mental health within general practices

The RPMW operated from within the general practice setting and coordinated care with other relevant healthcare providers across the first, second, and third lines of care. Within the practice, the RPMW played an accessible and supportive yet complementary role, becoming involved at the request of the GP when a patient presented with depressive symptoms.

Given the often limited consultation time available to GPs, there is little room to explore the full breadth of a patient's life context. In this respect, the RPMW helped to bridge a gap in care by supporting diagnostic exploration and assessing the patient’s needs using tools such as the OQ-45 as an aid where appropriate. Together, the GP and the RPMW formed a collaborative dyad that served as a central point of connection to other actors within the broader community and healthcare system.

As referring care professional

The RPMW was embedded in the local community and served as an expert on the regional health and social care landscape, making use of available online tools to ensure that patients could access appropriate support in a timely manner. This extended beyond purely psychotherapeutic care and, in line with a public health perspective, included a broader range of psychosocial support services.

The RPMW worked flexibly, guiding patients toward appropriate services related to lifestyle (e.g., nutrition, physical activity, smoking, sleep), socio-economic issues (e.g., housing, employment, education), and social connection (e.g., informal care, peer support).

As assertive care professional

The RPMW was not a mere referral agent whose role ended quickly. On the contrary, the RPMW acted as a *Compagnon de route*, a long-term ally in the patient’s journey, fulfilling an important signaling and support role. As a committed care professional, the RPMW helped guide the care trajectory, monitored progress, identified evolving needs, and routinely evaluated outcomes.

Routine Outcome Measurement (ROM) can be considered a tool for patient empowerment, potentially increasing patient engagement[4] and improving communication between care provider and patient[5]. Although existing evidence is limited[5-8], studies suggest a small effect of ROM on symptom reduction and treatment dropout[6,8], particularly among patients who might otherwise show no intervention effect[7,8]. Follow-up did not always have to occur in person; depending on the situation, telephone or video follow-up was a viable and recommended alternative[9].

The RPMW as supporter and motivator

Goal-oriented

The RPMW did not initiate a parallel therapeutic trajectory but did offer interim support to patients on waiting lists[10], an approach that can be perceived positively[11]. The RPMW acted as a motivating and supportive professional, without providing psychotherapy, yet offering targeted support on acute issues. Importantly, this was not framed as *"awaiting other care"*, as such phrasing could undermine the therapeutic climate. Instead, the RPMW provided meaningful, goal-oriented assistance during a critical period, reinforcing patient engagement and stability while navigating the broader care system.

Therapy enforcer

Healthcare is becoming increasingly digital. However, the use of digital apps without additional support is often associated with high dropout rates, and their clinical effectiveness remains debated. In contrast, digital apps with professional support have been shown to lead to clinically significant improvements[12,13]. Within the IDECA project, there was no intention to recommend a specific app for every patient. Instead, digital tools were only introduced when deemed appropriate, with the RPMW and patient jointly exploring suitable options.

The RPMW also engaged in social prescribing, encouraging patients to participate in activities such as nature-based programs, physical exercise (with or without a GP referral via *Bewegen op Verwijzing [Movement on Referral]*), and initiatives aimed at reducing social isolation—such as connecting with peers, community groups, or even animals[14].

While the prescribing of medication remained the sole responsibility of the GP or psychiatrist, the RPMW could play an important signaling and supportive role in pharmacological treatment. This was particularly relevant given that medication adherence among individuals with depression is often suboptimal[15]. The RPMW actively explored patients' expectations, experiences, and barriers (e.g., side effects), and communicated these insights to the GP. When medication was initiated or changed, it was essential to verify whether this had been thoroughly discussed with the patient and whether they felt fully informed.

The RPMW as information provider: self-management education modules

Integrated care emphasizes health literacy as a necessary condition for patient empowerment. In this context, *Ups & Downs* (a patient association for individuals with bipolar disorder or depression) collaborated with Janssen-Cilag to develop a depression information guide (Figure 1) [16].

The RPMW may deliver patient education to interested patients and their close ones. Recognizing that not every patient desires or requires the same kind of information, face-to-face educational

modules have been developed to explain key concepts related to depression, treatment, suicide, etc. Patients are invited to indicate which topics they are most interested in. They receive the guide to take home, allowing them to absorb the information at their own pace. During follow-up consultations, the RPMW proactively checks for understanding and addresses any questions.

The following six education modules have been developed:

1. Depression: what is it?
2. Treatment & support
3. Educational module for relatives
4. Suicide
5. Talking to your children
6. Life after depression

The education modules can be found online at OSF.

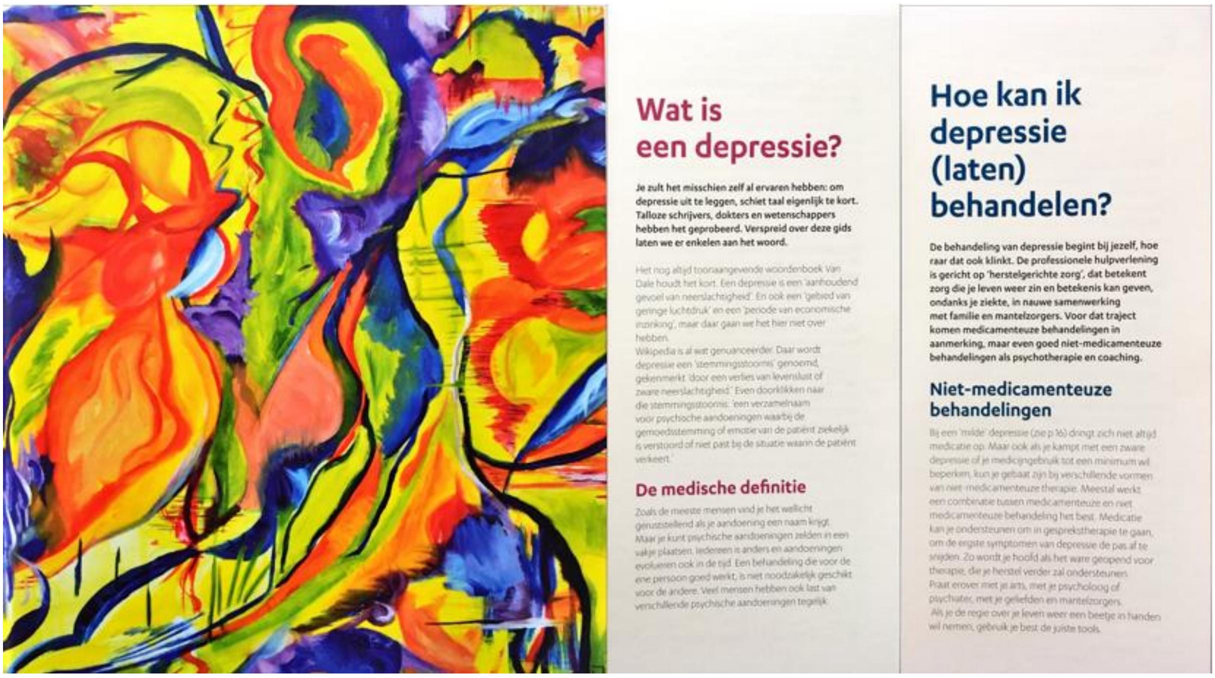

Figure 1. Information guide developed by Ups & Downs in collaboration with Janssen-Cilag. Left: cover. Right: extract.

Training of RPMWs

Between enrollment and first patient in, a training period of 6 weeks was foreseen to cover the content of Table 1. The training on the recovery- and goal-oriented communication techniques took place over a longer period of time.

Table 1. Training Content and Action Plan for RPMW Onboarding

Content	Trainer	Estimated Time

General training on the IDECA project	Research staff member (RW) + self-study at own pace	4 hours
Self-management educational modules on depression	Research staff member (RW) + self-study at own pace	3 hours (training) + 8 hours (self-study)
Module on Integrated Care	Research staff member (LB)	2 hours
Training on the use of monitoring tools (OQ-45-2)	Research staff member (RW) + self-study at own pace	1 hour
Recovery- and goal-oriented communication techniques<br>➢ Basic training at VIVEL site<br>➢ Advanced track with individual learning trajectory and peer review	Research staff member (RH)	Basic: 4 hours<br>Advanced: 8 hours<br>Peer review: 6 hours
Zipster Training (social prescribing digital tool; in PCZ MK)	Zipster staff member	2 hours
Regional care map and social services	PCZ staff member guided self-study	8–16 hours
Building relationships with general practitioners	RPMW-led	3–6 hours

General IDECA Project Training

The RPMW was expected to have read the full written intervention plan in advance. Project manager Ruben Willems has deliver a comprehensive PowerPoint presentation covering the entire project. This session has focused on the project's rationale, its various components, stakeholder roles and expectations, the RPMW's responsibilities and training trajectory, the shared care guideline and medication tools, the scientific evaluation design, and the project timeline. Ample time is provided for questions and discussion.

As part of the scientific evaluation, the session will explain the administrative procedures in detail: when and how to obtain informed consent; delivering signed forms to the project manager; administering the first questionnaire (online or on paper); maintaining a patient-specific log of contacts and activities using a standardized Excel template; and the timing of peer review sessions.

Depression education modules

The project manager has developed six patient education modules on depression, based on the *Depression Guide* and supplemented with recent scientific literature. These modules are designed for use in patient education sessions.

The training format depends on the background of the RPMW. The one who had a degree in clinical psychology and had ran a private practice, can independently work through the materials at own pace. The other RPMW, with a background in occupational therapy, expressed a need for more in-depth understanding of depression pathology. Therefore, in-person training had been provided.

Additionally, both RPMWs were expected to read the printed depression guide independently. For further information on depression, online resources such as www.depressiehulp.be and the Federal Health Portal's e-learning modules are used. These include courses on depression diagnosis and treatment, as well as on alcohol use and obesity.

Integrated care module

As the "I" in IDECA stands for Integrated, it is essential that RPMWs fully understand and embody the concept of integrated care. An expert in integrated care deliver a 2 hour online training session on this topic.

Monitoring tool

An important element in patient follow-up is the use of the validated routine outcome measurement tool OQ-45-2. This questionnaire is to be administered at the first contact and periodically thereafter. It can be completed by the patient in advance or during the session with the RPMW.

An Excel-based tool has been developed to calculate total and subdomain scores (social role functioning, distress, interpersonal relations), which can be compared against clinical cut-off values. Automated alerts are triggered for critical responses related to suicide, life satisfaction, and substance use. The RPMW has been trained to understand the three subdomains and how to interpret clinically relevant changes in scores.

Zipster training

The RPMW in PCZ Mechelen-Katelijne will have access to *Zipster*, a social prescribing platform. The developer of Zipster did provide a 1-hour information session based on this presentation. An online demo for self-study supplemented the information session: YouTube – How to use Zipster.

Regional Care and Social Map

For both RPMWs (especially in the context of Zipster), it is essential to become familiar with the local social and healthcare landscape. With the help of the staff member from their respective PCZs, RPMWs did compile a comprehensive overview of available services across all levels of care, including recovery colleges, psychologists, dietitians, and, importantly, services in the zero-line such as walking groups, sports clubs, and hobby clubs.

Building relationships with GPs

The RPMWs did visit all participating GP practices to meet GPs and colleagues in person and to familiarize themselves with the physical setting. This initial round of visits, following an online introduction, served as an opportunity to establish working relationships and agree on collaboration practices.

Recovery and Goal-oriented care

Goal-oriented care (GOC) shifts the focus from disease and problem-oriented care to what is meaningful to the person. At the level of the clinical encounter, GOC offers a practical framework for aligning care plans with personal goals rather than with condition-specific protocols, professional routines, or organizational workflows. At the team and organizational level, GOC provides a unifying philosophy for primary care providers by creating shared goals rooted in the patient's priorities.

Within the GOC research group at Ghent University, Valentijn's Rainbow Model of Integrated Care[17] is used as a guiding framework for designing GOC interventions. The framework posits that when an innovation is introduced to facilitate integrated care, its implementation must address and balance functional and normative integration. Functional integration concerns the practical conditions and enablers that determine how the innovation can be carried out (e.g., use of a shared electronic record). Equally important is normative integration, which focuses on developing a shared vision and common goals (e.g., strengthened cohesion and collaboration among care actors).

GOC training had two objectives. First, competency development in RPMWs. RPMWs were supported to provide care in which personal goals guide assessment, planning and follow-up. Second, facilitation of team and system collaboration. RPMWs, GPs and other health and social care professionals learned together how the RPMW role can enhance interprofessional collaboration and contribute to integrated care for people supported through an RPMW.

To meet these objectives, two parallel pathways were implemented:

- Individual pathway (RPMW-centered): tailored to each RPMW's needs and experiences. This pathway contained three training sessions with the two RPMWs to exchange experiences and build skills.
- Local collective pathway (PCZ-centered): tailored to the primary care zone (PCZ) where the innovation was being implemented. This pathway contained three evening peer-supervision sessions in the respective PCZs, involving the RPMWs and colleagues from local GP practices.

Individual learning pathway (RPMW-centred)

Preparation: Each RPMW completed VIVEL knowledge-clip modules on Goal-Oriented Care (GOC).

TRAINING SESSION 1 – FOUNDATIONS & ORIENTATION	
Date	17 April 2023 (during 6 week training period of RPMWs)
Aims	• Introductions, expectations and motivation for taking on the new RPMW role.<br>• Positioning GOC as a supporting framework.
Content	**Opening round:** Short introductions and articulation of individual learning aims.<br><br>**Mini-lecture with visuals:** Concise slide set framing GOC (key principles, language of goals, iterative review).<br><br>**Facilitated dialogue (four themes):**

	• From a good conversation to personal goals.<br>• Combining areas of expertise: balancing therapeutic skills with the RPMW function.<br>• GOC is not a solo act: the value of collaboration.<br>• Realising and reviewing goals over time

TRAINING SESSION 2 – FIRST EXPERIENCES, TOOL AND NETWORK MAPPING	
Date	1 June 2023
Aims	• Surface early experience through a real case.<br>• Map the RPMW's collaborative network.<br>• Explore practical tools for goal-elicitation conversations.
Content	**Case bring-in:** One RPMW presented a current case (de-identified).<br><br>**Stakeholder mapping:** Guided exercise to map partners across health and social care (who is involved, who is missing, roles, information flows).<br><br>**Tool walk-through:** Brief demonstrations of GOC-aligned tools (goal elicitation prompts, goal- setting tools)<br><br>**Short theory refreshers:**<br>• From "what's the matter?" to "what matters to you?".<br>• Patient autonomy and the skilled companionship stance.<br>• Motivation via perceived control and acceptance (Bloem & Stalpers).<br>• Supporting self-management.

TRAINING SESSION 3 – ROLE DELINEATION & COMPETENCY PROFILING	
Date	29 February 2024
Aims	• Use recent cases to clarify competencies that are distinctive to—or complementary within—the RPMW role.<br>• Inform future profile delineation and development priorities.
Content	**Two case exemplars:** Each RPMW selected a "school-example" case where RPMW input seemed particularly valuable.<br><br>**Guided prompts:**<br>• Tasks undertaken by the RPMW in each case.<br>• Which tasks are intrinsic to RPMW vs could be undertaken by others?<br>• Interventions specific to RPMW (vs GP, primary-care psychologist, social worker) and the rationale.<br>• How RPMW approach differed from first- or second-line professionals.<br>• Personal and professional qualities that proved most helpful.<br>• Skills/knowledge gaps and desired upskilling areas.<br><br>**Competencies of the RPMW:** The T-shaped professional model was used to separate generic (broad) from specialist (deep) competencies. Revisited the team-coined analogy "from deep-sea diver to octopus" to balance depth of

	expertise with wide navigation across services—guarding against designing a role for exceptionally rare competency profiles.

Local collective learning pathway (PCZ-centred)

Each supervision session was structured by the Normalization Process Theory (NPT) to track implementation of the RPMW over time:

- Coherence — shared understanding of the intervention and its purpose.
- Cognitive participation — collective buy-in and willingness to invest effort.
- Collective action — the work done to enact and sustain the innovation.
- Reflexive monitoring — appraisal of effects, value and impact on people and practice.

PEER-SUPERVISION SESSION 1	
Dates	PCZ Mechelen-Katelijne: 22 Juni 2023; PCZ Voorkempen: 28 September 2023
Aims	• Debate perspectives and expectations on the innovation across the local team.<br>• Identify practical barriers affecting RPMW work (referral, communication, follow-up).<br>• Build a first shared view—via a case—of where RPMW adds value.
Content	**Warm-up alignment:** Brief activity capturing each participant's *vision and expectations* for the RPMW role (from both the selected RPMW and involved GPs).<br><br>**Needs assessment (two lenses):**<br>• **RPMW needs** to enact the role and meet expectations.<br>• **GP/practice needs** for collaboration with an RPMW (roles, communication, referral cues).<br><br>**Case-anchored dialogue and exemplar questions:**<br><br>**1) Access & referral**<br>• Looking back over the past months, who do you consider suitable for an RPMW trajectory? Who was referred, who was not, and why in each case?<br>• How are referral decisions made? What triggers (signals/cues) lead you to refer to the RPMW?<br><br>**2) Role delineation**<br>• Who takes which role at each stage of a person's support pathway? Where does the GP's remit end, and from when is RPMW involvement warranted?<br>• How do you view the RPMW role versus the in-practice psychologist's role? What is the distinction between referring to the RPMW and to the psychologist in your practice—when, why, and on what grounds (GP/RPMW/psychologist/other colleagues)?<br><br>**3) Follow-up & process management**

<table>
<tr><td></td><td>

How do you envisage process guidance and follow-up for patients supported by an RPMW? What shared agreements are in place during such episodes?
How should follow-up be organised and communicated? Which communication strategies (e.g., shared planning, debriefs after patient contact) would you use, and what is needed to put these in place?

4) Project outlook

Fast-forward to the end of the project: what changes do you hope to see in your practice and in your work?
What will be visibly different—what, for whom, and how? For you, for patients, and for the practice/team?

</td></tr>
</table>

<table>
<tr><th colspan="2">PEER-SUPERVISION SESSION 2</th></tr>
<tr><td>Dates</td><td>PCZ Mechelen-Katelijne: 16 November 2023; PCZ Voorkempen: 18 January 2024</td></tr>
<tr><td>Aims</td><td>

Start from the desired change stakeholders seek to achieve through the RPMW innovation, and translate this into concrete, practicable actions that facilitate RPMW implementation.
To situate actions within Valentijn's Rainbow Model of Integrated Care, ensuring attention to micro (patient–provider), meso (team/organisation), and macro (system/zone) levels.

</td></tr>
<tr><td>Content</td><td>
Themes and exemplar questions if the session:
Embedding the RPMW innovation in primary care

How long should an RPMW episode run—what scope/duration, and is it finite by design?
When is discharge appropriate vs assertive outreach, and which personal/context factors make "letting go" difficult?

Handover and network reach

During/after an RPMW episode, to whom do we refer or hand over—and when?
Which partners are we reaching vs missing, and what is the impact on patients, the RPMW, and the GP practice?

Planned outputs

A brief episode definition (scope, ceiling, closure criteria, outreach parameters).
An action list mapped to micro/meso/macro levels (owners, timelines), plus a referral/hand-over matrix and network gap list.

</td></tr>
</table>

<table>
<tr><th colspan="2">PEER-SUPERVISION SESSION 3</th></tr>
<tr><td>Dates</td><td>PCZ Mechelen-Katelijne: 5 September 2024; PCZ Voorkempen: 24 October 2024</td></tr>
<tr><td>Aims</td><td>

Review interim economic evaluation findings (project lead presentation).
Delineate patient profile(s) best suited for RPMW support.

</td></tr>
</table>

	• Conduct a **SWOT analysis** of RPMW implementation.
Content	**Findings briefing:** Short presentation with Q&A; implications harvested on sticky notes.<br><br>**Profile delineation:** Grouping recent RPMW cases to identify shared characteristics (complexity, comorbidity, social determinants, motivation).<br><br>**SWOT workshop:** Small-group rotations on Strengths, Weaknesses, Opportunities, Threats; plenary synthesis into priorities.<br><br>**Strengths**<br>• What advantages does the RPMW bring to patients, the GP practice, and the ELZ? Which of our team/organizational assets enable successful RPMW implementation?<br>• What positive feedback have we received (patients/GPs/colleagues)? In which case types did the RPMW add most value?<br><br>**Weaknesses**<br>• Which internal factors (communication, capacity, skills, workflows, IT/EHR) hinder RPMW work or coordination?<br>• Which persistent problems remain unsolved despite RPMW involvement, or where are we inefficient?<br><br>**Opportunities**<br>• Which collaboration opportunities with other organizations or stakeholders could strengthen RPMW pathways? Who is missing from our network and how can we reach them?<br>• Could/should the scope of the RPMW extend (e.g., beyond GP practices or to defined patient segments)? Which favourable trends/policies could we leverage?<br><br>**Threats**<br>• What external risks could negatively affect RPMW implementation (e.g., regulation, funding, workforce competition/role overlap, data-sharing constraints)?<br>• Are there practice-level risks in employing an RPMW (sustainability, caseload ceilings, liability), or organisational weaknesses that impede integration?

Together, the individual and local–collective pathways provided sustained, practice-embedded support: building RPMW competencies, clarifying role boundaries, and strengthening interprofessional working in each PCZ.

2. Shared care guideline

The IDECA intervention developed a cross-level shared care guideline for general practitioners (and RPMW), based on and complementing the existing Belgian Domus Medica guideline for the treatment of depression in adults[18]. Crucially, this document serves as a supportive tool rather than a prescriptive protocol.

The IDECA intervention, including the role of the RPMW, has been integrated into the guideline. The guideline takes the form of a visual “eye-catcher” that outlines the diagnostic process, the most common care elements, and referral principles across the zero, first, second, and third lines of care, all presented in the form of a decision tree.

The guideline has been validated by a scientific steering committee which has gathered multiple times. The visualized guideline has been printed on A3, and has been distributed online. Figure 2. Shared care guideline (in Dutch). A PDF version in better resolution is accessible on OSF.Figure 2 shows a screenshot but a better PDF resolution can be found on OSF[19].

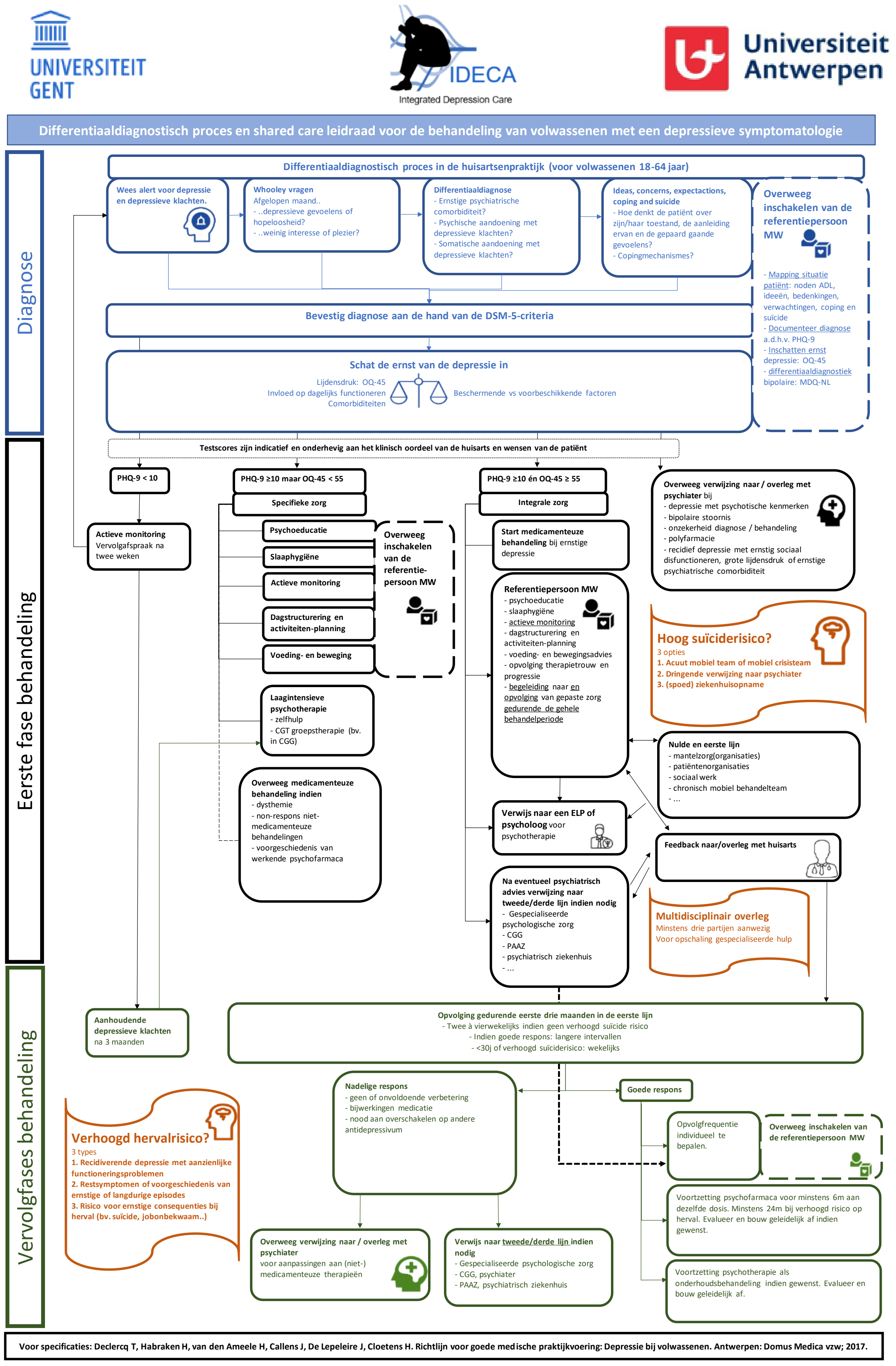

Figure 2. Shared care guideline (in Dutch). A PDF version in better resolution is accessible on OSF.

3.Medication pathway

The visualized decision tree for prescribing medication, developed for general practitioners and based on the existing Domus Medica guideline for the treatment of depression in adults[18], can be found in Figure 3 and in a better resolution on OSF[19]. The medication pathway has been validated by two psychiatrists.

The document has been printed in A3 format and has been made available digitally.

It is essential to emphasize that this tool serves as a guideline, a helpful reference, but not a prescriptive rule. Every patient is unique, and what works for one individual may not work for another, even when clinical characteristics are similar. Often, treatment involves a process of trial and error, which becomes particularly evident when actions such as dose increases, switching antidepressant classes, combining different classes, or applying augmentation strategies are considered in response to a lack of or inadequate therapeutic effect from the initial pharmacological treatment.

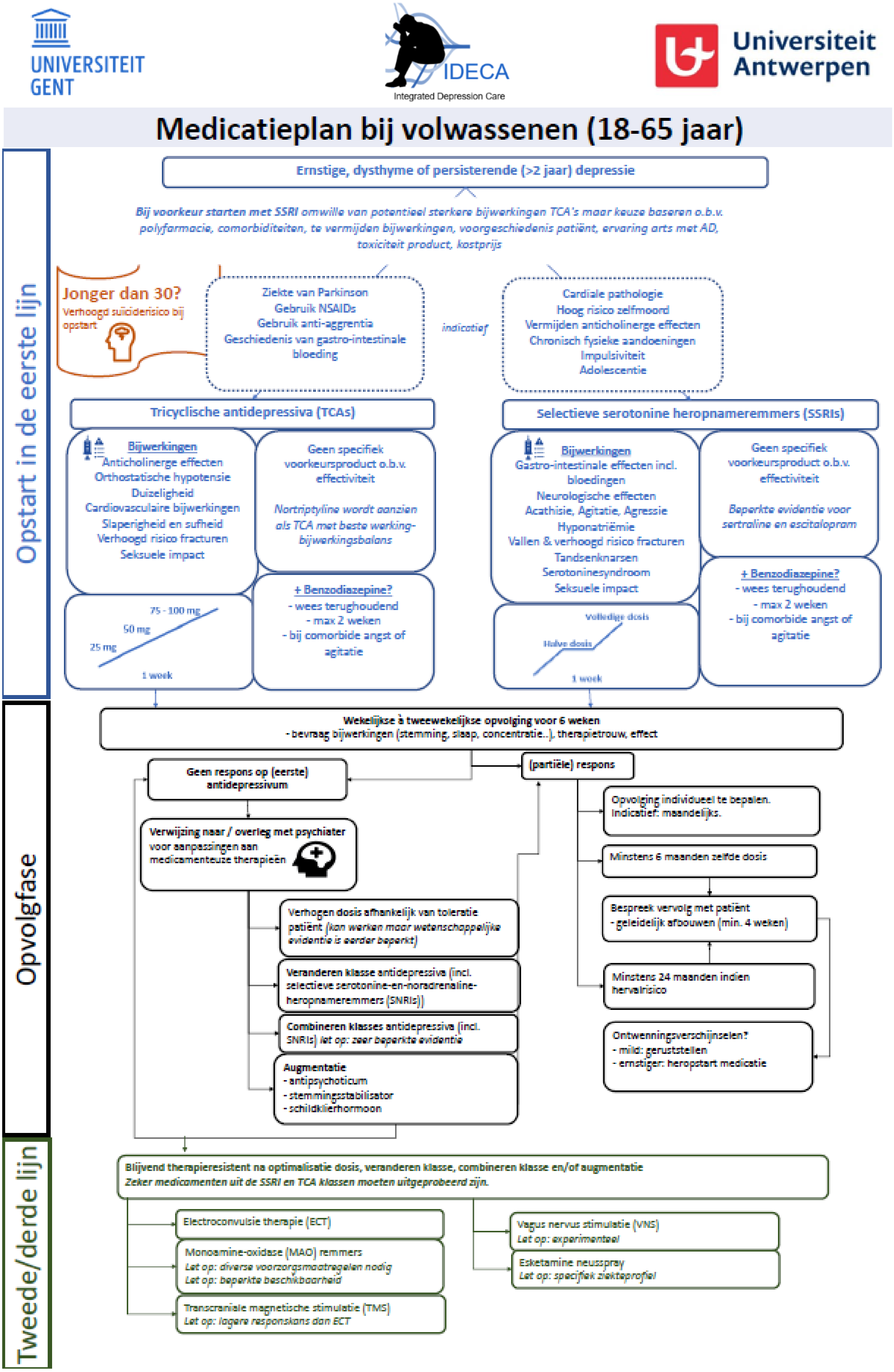

Figure 3. Medication path (in Dutch). A PDF version in better resolution is accessible on OSF.

4.GP training

The refresher training addressed the care for individuals with depressive symptomatology from an integrated care perspective, emphasizing the foundational role of strong primary care in delivering appropriate cross-level support. The two-hour session was co-moderated by integrated care expert Liesbeth Borgermans and project manager Ruben Willems. It covered the following topics:

(i) an introduction to depression by Kris Van den Broeck,
(ii) population management for depression by Geert Goderis,
(iii) cross-level shared care from the perspective of general practice by Kris Van den Broeck, and
(iv) medication management by Didier Schrijvers.

The trainings took place on May 11 and May 25 in PCZ Mechelen-Katelijne and PCZ Voorkempen, respectively. Accreditation points were requested and approved.

Presentations can be found on OSF[19].

Research design

Single arm implementation

The IDECA study adopts a single arm implementation design. As a result, the benefits of randomization are entirely absent, and no adjustments can be made for the effect of covariates. Consequently, any clinically significant improvements cannot be attributed to the IDECA intervention itself for sure; assessing the intervention's effectiveness is therefore not the main objective of this study. Nevertheless, an implementation study without a control arm is considered the most appropriate approach for several reasons:

(i) The IDECA project should be understood as a proof-of-concept study, in which the experiences of both healthcare providers and patients are pivotal. The primary objective is to identify which aspects of the intervention are perceived positively and which require adjustment in view of a potential scale-up to a quasi-randomized trial with adequate resources.
(ii) In a quasi-randomized design, selection bias would inherently arise due to bias by indication: the control group would, by definition, not have access to integrated care, whereas in the intervention group, inclusion would partly be based on a subjective assessment by general practitioners of whether such care is needed—thus compromising comparability.
(iii) The power of the IDECA proof-of-concept study is inherently limited, as the implementation of a RPMW is a resource-intensive intervention. Personnel costs are traditionally a major budgetary burden, and the number of patients included is expected to remain modest, resulting in limited statistical power.
(iv) Previous research indicates that in such integrated care interventions, it is particularly challenging to avoid high dropout rates in the control group, which carries substantial statistical implications.

Heterogeneous patient population calls for a real-world approach

Several considerations must be taken into account when defining the patient population. First, there is no single, uniform profile of a patient presenting with depressive symptomatology (see also Figure 4)[20]. The heterogeneity among individuals with depression is substantial; it is entirely possible for two patients who both meet DSM-5 diagnostic criteria to share only minimal overlap in terms of symptomatic domains. While the DSM provides a useful diagnostic framework, relying solely on DSM criteria for patient inclusion is not recommended.

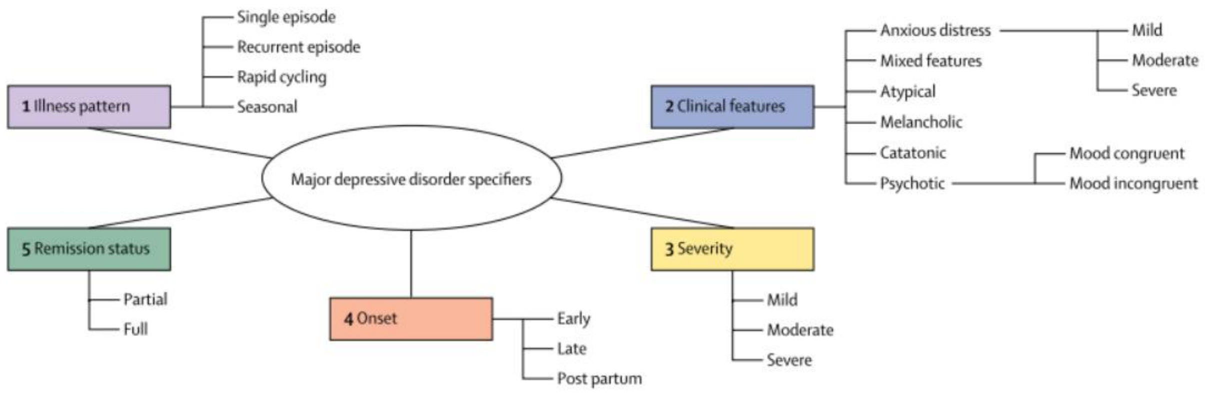

Figure 4. Major depressive disorder specifiers. reproduced from Malhi (2018)

Secondly, the target population is characterized by a high degree of comorbidity, and the external validity of the study would be compromised if overly strict exclusion criteria were applied. In this regard, the primary aim of the study must be taken into account: this is a proof-of-concept project intended to align as closely as possible with real-world clinical practice and to be acceptable to clinicians. Given the absence of a control group, the importance of inclusion and exclusion criteria is further diminished.

Thirdly, a balance must be struck between the healthcare provider's subjective assessment and the objective evaluation of symptomatology and resulting patient needs. This is a complex task due to the aforementioned heterogeneity and the multidimensional functional impairments commonly observed in patients with depressive symptoms.

Taking these arguments into account, the patient population for the IDECA intervention is intentionally defined in broad terms: all patients who are diagnosed with depression through the differential diagnostic process in general practice are eligible for inclusion. The criteria for identifying depression are as follows:

- **Primary screening**: The patient must have responded positively to at least one of the two Whooley questions, which have a reported sensitivity of 97% and specificity of 67%[18]:
    - *During the past month, have you often been bothered by feeling down, depressed, or hopeless?*
    - *During the past month, have you often been bothered by little interest or pleasure in doing things?*
- **DSM criteria**: The Patient Health Questionnaire (PHQ-9)[21], which is DSM-based, may be used as an indicative tool for depression diagnosis. However, it is not sufficient on its own to establish a definitive diagnosis, and should therefore be considered as a guiding

instrument. A meta-analysis showed that a cutoff score of 10 is associated with a sensitivity of 0.86 (95% CI: 0.80–0.90) and a specificity of 0.85 (95% CI: 0.82–0.87)[22].

When deemed appropriate by both the patient and the general practitioner, the RPMW is involved to conduct a broader assessment across various life domains. In close collaboration with the patient and the treating GP, the RPMW determines whether a specific intervention (purely therapeutic or pharmacological) or a more comprehensive, integrative intervention is needed (see also 'Shared Care Guideline').

With the exception of age (patients under 18 and over 65 are excluded), no strict exclusion criteria have been applied. However, the Shared Care Guideline outlines situations in which involving the RPMW may not be advisable as an initial step and where immediate referral to specialized services or providers is recommended. In line with the Domus Medica guideline, this includes the following situations:

- High suicide risk;
- Bipolar depression;
- Postpartum depression;
- Depression with psychotic features;
- Diagnostic uncertainty or uncertainty about treatment initiation;
- Polypharmacy.

This did not preclude a role for the RPMW at a later stage of the care trajectory.

Additionally, referral to or consultation with specialized services is also advised under the Domus Medica guideline in the following circumstances:

- Insufficient improvement or non-response to the initiated treatment;
- In case of side effects from medication, or when switching antidepressants or using combinations of medications;
- Recurrent depression with significant social dysfunction, high psychological distress, or severe psychiatric comorbidity.

Intervention area recruitment

The IDECA research group contacted the Flemish Institute for First Line (Vlaamse Instituut voor de eerste lijn; VIVEL) in early and spring 2022 to present the IDECA project and to call for their help in recruiting primary care zones (PCZ). A primary care zone is a network of primary care providers in a geographically defined area. They exchange knowledge and information with each other and coordinate operations. They also look at local care and welfare needs to improve the quality of health care. A PCZ is a geographically defined are consisting of one or more cities or towns, controlled by a healthcare council, is a network of primary care providers, and has at least 60,000 inhabitants. Flanders and Brussels is divided into 60 of those PCZs.

In June 2022, two online information sessions were held at which about 15 PCZs participated. Seven PCZs applied formally by July 14th to participate in the IDECA project. September and October 2022 were used to have bilateral talks with each PCZ, after which two were selected. Selection criteria were (i) motivation to be advocates of the IDECA project, (ii) ability to mobilize

about 10 GPs, (iii) motivation to engage in a co-create interaction with the RPMW to train this person in becoming an expert of local social and health providers, (iv) participation of mental health actors in the healthcare council, and (v) having an idea who could become the RPMW. The two selected PCZ were instructed to recruit about 10 general practitioners (GPs) working in different settings (e.g., multidisciplinary practice, solo practitioner), resulting in 7 general practices with at first 23 GPs in total (Figure 5). Not all GPs participated equally active throughout the intervention period, and some were GPs trainees who could not participate for the whole intervention period.

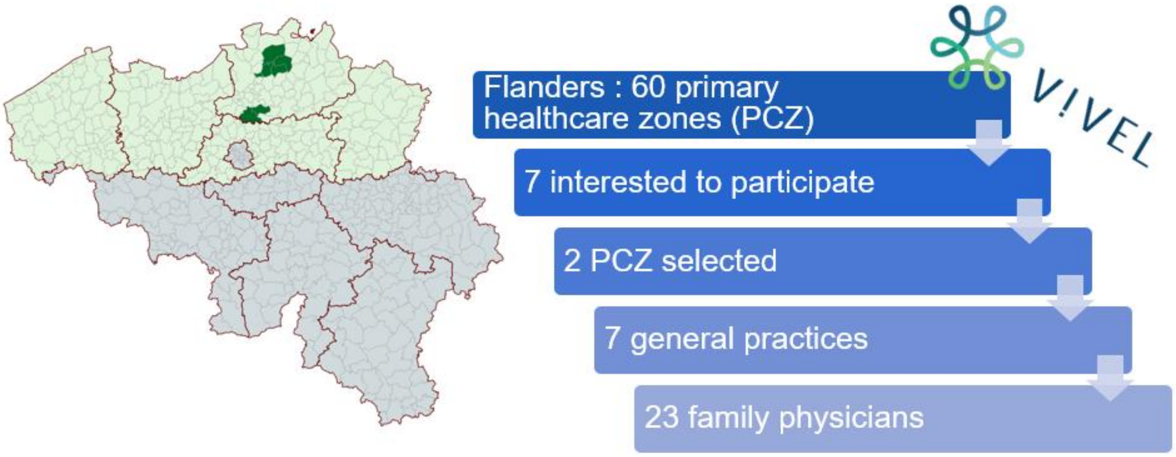

Figure 5. Intervention area recruitment process.

Intervention areas

The IDECA intervention has been implemented in two PCZs. PCZ Mechelen-Katelijne, staffed by three project workers, serves a total population of 108,523 inhabitants (as of 2021), with a population density of 1,065 inhabitants per km^2, which is substantially higher than the average population density of 489 per km^2 in the Flemish Region. However, there is a significant difference between the urban area of Mechelen and the rural municipality of Sint-Katelijne-Waver. Compared to the Flemish Region, the area has relatively more young people and people of working age, and fewer older adults. The average socio-economic status of the inhabitants is representative of the Flemish Region. Respectively 83.5% and 85.6% of eligible residents in Mechelen and Sint-Katelijne-Waver have a general medical record, which is higher than the Flemish average of 82.2%. In Mechelen, there are 79 general practitioners (GPs) working in solo and primarily group practices, as well as a Community Health Centre; in Sint-Katelijne-Waver, there are 5 practices[23].

PCZ Voorkempen serves a total population of 110,795 inhabitants (as of 2021), with a population density of 416 inhabitants per km^2, slightly lower than the average of 489 per km^2 in the Flemish Region. However, there are substantial differences between municipalities, ranging from Malle (301/km^2) to Wijnegem (1,280/km^2). Compared to the Flemish Region, the area has relatively fewer young people and working-age individuals and more older adults, indicating a real ageing pressure. The average socio-economic status of the residents is relatively representative of the Flemish Region, with slightly more people employed and a smaller share of non-working individuals. In Voorkempen, there are 91 active GPs, supplemented in 2021 by 18 GPs in training[24].

Participating general practices

In PCZ Mechelen-Katelijne, four general practices participated with each having distinct organizational structures and multidisciplinary collaborations. Descriptions reflect the situation at intervention start but could have evolved throughout the intervention period:

(i) a solo GP practice that operates within a multidisciplinary setting. It includes part-time collaboration with a nurse (two half-days per week), a psychologist with a specialization in addiction, a medical pedicurist, and a speech therapist—all of whom work on a secondary employment basis.

(ii) a group practice with three GPs, supported by practice assistants and a nurse responsible for daily blood draws in a dedicated lab space each morning. The team also includes a psychologist, for whom this is the primary workplace, and a coach specializing in stress and burnout who is available two half-days per week.

(iii) a large group practice with seven GPs, of whom five will actively participate in the study and one will be partially involved. The non-active and semi-active GPs are mainly based at a different location. Two of the active GPs are trainees: one did complete training in March 2024, and one already in May 2023 but the latter planned to remain working in the practice. The practice includes four consulting rooms for GPs and four rooms for other health professionals. A wide range of other professions are represented in the practice, each using the facilities on a part-time basis.

(iv) A group practice with four GPs, supported by one GP trainee, and two nurses of which one acting as a diabetes educator.

In PCZ Voorkempen, the following three practices contributed to the project with diverse teams and varying scopes of interdisciplinary care.

(i) a medical center staffed by two GPs and a BOV (movement on referral) coach. The team is set to expand, with a personal coach joining in April 2023 and a primary care psychologist starting in November 2023, further strengthening its multidisciplinary approach.

(ii) a group practice consisting of four GPs and two GP trainees. The team also included a full-time physiotherapist, a part-time children's coach, and a dietitian who rented space on a part-time basis. Additionally, there was a psychotherapist affiliated with the practice, although back then temporary unavailable.

(iii) a center featuring one GP and one GP trainee who was present for the entire duration of the project. The center hosts two psychologists who work with both children and adults. The team also includes a nurse specialized in blood draws.

Selection of the RPMW

The RPMW has been discussed before under 'intervention components'. In PCZ Mechelen-Katelijne, an open vacancy was published for the position of RPMW, followed by a structured selection process consisting of three rounds. Ultimately, a candidate with an educational background in clinical psychology was appointed. In contrast, in PCZ Voorkempen, the local care council proposed a candidate who was motivated to take on a new professional challenge. After an interview with the research team, this candidate, holding a degree in occupational therapy and having previous professional experience in case management, was selected for the role.

Evaluation tools

IDECA did apply a mixed-method research methodology (Figure 6).

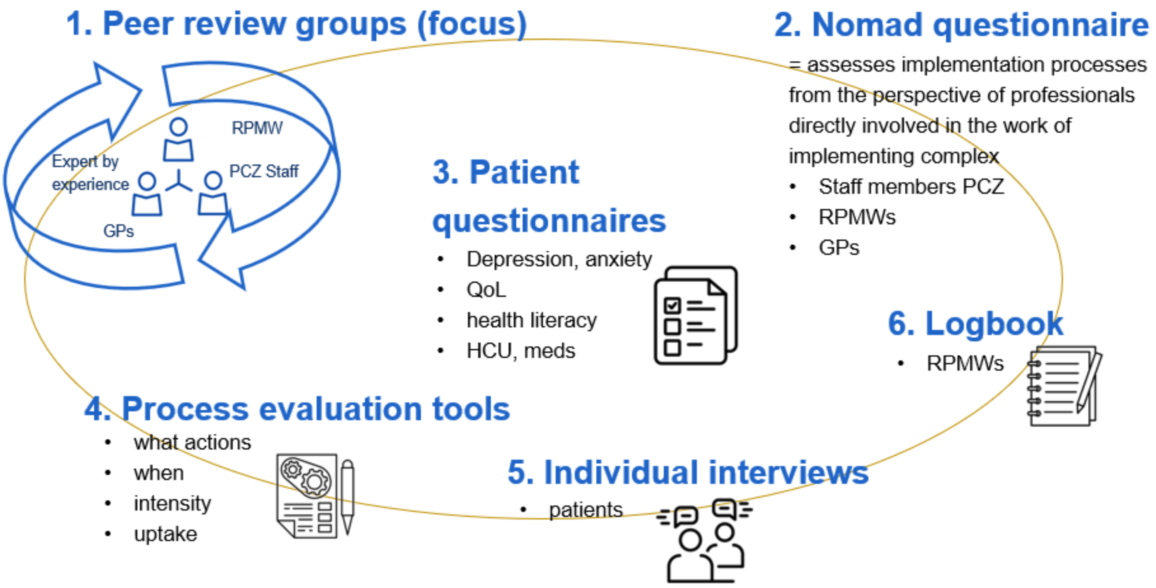

Figure 6. Overview mixed-method evaluation tools.

Quantitative: patient questionnaires

Patients were surveyed at five different time points (at inclusion and after 3, 6, 9, and 12 months). At inclusion, the questionnaire was provided on paper either by the general practitioner or the RPMW. Patients also had the option to complete the questionnaire directly online, but this option was rarely used (N=5). Invitations for the follow-up questionnaires were sent via email and completed digitally through Qualtrics, with four reminders sent at one-week intervals. Patients who had their first contact with the RPMW between the start of the intervention at the end of April 2023 and December 31, 2023, were invited to complete the follow-up questionnaires.

In addition to a brief demographic questionnaire at inclusion, the following aspects were assessed using corresponding questionnaires:

- Depression: Outcome Questionnaire-45[25,26] en Patient Health Questionnaire-9[21,27-29];
- Anxiety*: Outcome Questionnaire-45[25,26];
- Health literacy**: HLS-EU-Q16[30,31];
- Patient satisfaction**: Europep[32];
- Quality of life: EQ-5D-5L en EQ VAS scale[33];
- Medication use: MARS to assess therapy adherence[34-36], MUSE to assess self-efficacy to understand and apply medication instructions[37];
- Healthcare utilization: shortened iMCQ, extended by targeted questions on absenteism[38].

*Only administered at T0 and T4 (and clinical-wise: whenever the RPMW thought it suited)

**Only administered at T0, T2 and T4

Quantitative: NoMad

To map the implementation process from the perspective of the healthcare providers, a modified version of the NoMAD questionnaire was used[39], which can be consulted on OSF[19]. This questionnaire was developed to gain a better understanding of how new technologies and complex interventions are adopted and integrated into healthcare settings.

Part A explores the respondent's background, Part B contains general questions about innovation normalization and process factors, and Part C includes detailed questions focused on the four core processes of Normalization Process Theory (NPT): coherence, cognitive participation, collective action, and reflexive monitoring. The NoMAD was administered every four months: T0 at the start (after 2 weeks), T1 after 4 months, T2 after 8 months, T3 after 12 months, and T4 after 16 months. Responses are converted into Likert scales.

Qualitative: focus (peer review) groups

The professional profile of the RPMW was defined through a qualitative analysis of two peer review sessions conducted in each of the two respective PCZs where the IDECA project was implemented. This served as a complement and refinement of the initial framework outlined in the IDECA intervention plan.

Within these same peer review sessions, the patient profile referred by general practitioners was also discussed. Although the shared care guideline defined a specific patient profile thought to benefit most from referral to the RPMW, it was emphasized to the GPs that this was a non-prescriptive guideline. The patient group was intended to be defined from the bottom up.

The peer review sessions of 90–120 minutes each were organized to further develop the role of the RPMW. The first peer review sessions took place approximately 2 and 4 months, and the second sessions approximately 6.5 and 7.5 months after the respective first patient inclusions in PCZ Mechelen-Katelijne and PCZ Voorkempen. The second peer review in PCZ Voorkempen was held digitally due to unforeseen railway disruptions.

The first peer review aimed to map out possible scenarios of a care trajectory for a person being supported by the RPMW, and to identify the conditions required to support such a trajectory effectively. The peer review guide was structured around four topics:

(i) intake, which patients were referred to the RPMW and why,
(ii) division of roles in the support process,
(iii) follow-up of patients during the trajectory, and
(iv) views on how the project could impact daily practice.

The second peer review focused on actively integrating the RPMW into care. The peer review guide was structured in two parts:

(i) the course of the care trajectory, with a focus on assertive care and discharge, and
(ii) collaboration and referral between the RPMW and the GP, as well as with other healthcare providers in the care landscape.

Each peer review included the RPMW, the involved general practitioners, and the staff member from the PCZ. There was no requirement for all GPs to attend, but each participating GP practice was asked to send a representative. The sessions were facilitated by at least two members of the

scientific team from Ghent University. All peer review sessions were recorded, transcribed, and thematically analyzed (Table 2).

Table 2. Attendees at peer review sessions.

	Scientific team	RPMW	GPs	Staff members
PCZ MK – Peer review 1	4	1	3	1
PCZ VK – Peer review 1	2	1	4	1
PCZ MK – Peer review 2	2	1	3	1
PCZ VK – Peer review 2	3	1	3	1

A third peer review session was planned as well but was postponed at the request of the RPMWs until the final phase of their appointments (18 months after first patient inclusion). This third peer review session was used as a member check of first analyses on RPMW professional profile, patient profile, case load, and budget impact.

More details of the peer review sessions can be found above under Recovery and Goal-oriented care – Local collective learning pathway.

Qualitative: individual interviews

On September 20, 2024, ten patients from each Primary Care Zone (ELZ) were invited via email to participate in a semi-structured interview. A reminder email was sent on October 1, 2024, after which six individuals responded positively. On November 13, another fifteen patients were invited, resulting in two additional positive responses. This yields a response rate of 8 out of 45 invited patients (out of a total of 201 patients who had been seen).

A semi-structured interview guide covering four main themes was used (Table 3). Questions explored:

(i) the course of depressive symptoms,
(ii) expectations and experiences regarding the RPMW (Remote Patient Monitoring for Wellbeing),
(iii) psychoeducation, and
(iv) perceived gaps in the healthcare system.

Interviewers actively asked for examples and context to properly interpret participants' responses. The interviews were conducted by Ruben Willems or Liesbeth Borgermans. All interviews were recorded and transcribed. The aforementioned researchers performed the analysis, following the guidelines of Braun & Clarke[40] for phenomenological thematic analysis. Phenomenological research examines participants' lived experiences through their subjective, first-person accounts[41]. The analysis process resulted in a thematic tree with main themes and subthemes. In this study, themes were strongly inductively derived based on the interview guide.

English translation (by artificial intelligence) of the original Dutch interview guide:

Table 3. Semi-structured interview guide, freely translated from Dutch to English.

QUESTIONS	NOTES<br>**Target interview duration: 30–60 minutes**
**Depression**	
• How did you experience the treatment of your depression / how are you currently experiencing it?	*Follow up to understand the experience, potential comorbidities, and actions taken.*
**Reference Person Mental Wellbeing (RPMW)**	
• What were your expectations when your GP referred you to the RPMW?<br>o Which expectations were met?<br>o Which expectations had to be adjusted?	*Reflect on each expectation.*
• How did you experience the contact with the RPMW?<br>o What were the crucial factors that made the RPMW feel helpful or unhelpful to you?	
• How did you experience the collaboration between the RPMW and other healthcare professionals?	*Pay attention to each collaboration mentioned.*
• Which competencies are crucial for the RPMW?<br>o Which competencies were lacking and should be addressed through training?<br>o How should the RPMW role be further developed?	
**Psychoeducation**	*Not all participants received psychoeducation, so this may not apply to everyone.*
• Which modules of the psychoeducation were relevant to you?	
• In your opinion, which modules are still missing?	
**Healthcare landscape**	
• Which healthcare professionals or services in the current healthcare system do you use, and why?	
• Which healthcare professionals or services do you feel are missing?	

- How do you see the future evolution of the general practice?
    - What does your ideal general practice look like?

- In what way is the RPMW a valuable addition to the existing healthcare landscape?

Process evaluation

RPMWs were asked to make notes of each encounter with a patient. They reported date, session length, and what the session was about in keywords. The process evaluation document was filled out for the full 18 months and for every patient seen.

Logbook

The RPMWs kept a detailed activity log over a two-week period—April 15–26 for the RPMW in PCZ Mechelen-Katelijne and May 27–June 7, 2024 for the RPMW in PCZ Voorkempen. In this log, activities were recorded minute by minute, specifying the type of activity (e.g., patient contact, preparation for patient contact, follow-up work after patient contact, in-person contact, telephone contact, travel time, administration, meetings, training, consultation with GP, consultation with other healthcare professionals). Additional context or elaboration could be provided where relevant. The logbook was discussed in advance with the RPMWs.

References

1 Vlaamse-Overheid. IKW Chronische ziekten - Gemeenschappelijk Plan Geïntegreerde Zorg - Gemeenschappelijke visie over zorgcoördinatie en casemanagement. (Vlaamse Overheid, Brussel, 2019).

2 Bower, P., Gilbody, S., Richards, D., Fletcher, J. & Sutton, A. Collaborative care for depression in primary care. Making sense of a complex intervention: systematic review and meta-regression. *Br J Psychiatry* **189**, 484-493 (2006). https://doi.org:10.1192/bjp.bp.106.023655

3 Moise, N. *et al.* Interventions to Increase Depression Treatment Initiation in Primary Care Patients: a Systematic Review. *J Gen Intern Med* **33**, 1978-1989 (2018). https://doi.org:10.1007/s11606-018-4554-z

4 Solstad, S. M., Castonguay, L. G. & Moltu, C. Patients' experiences with routine outcome monitoring and clinical feedback systems: A systematic review and synthesis of qualitative empirical literature. *Psychother Res* **29**, 157-170 (2019). https://doi.org:10.1080/10503307.2017.1326645

5 Carlier, I. V. *et al.* Routine outcome monitoring and feedback on physical or mental health status: evidence and theory. *J Eval Clin Pract* **18**, 104-110 (2012). https://doi.org:10.1111/j.1365-2753.2010.01543.x

6 de Jong, K. *et al.* Using progress feedback to improve outcomes and reduce drop-out, treatment duration, and deterioration: A multilevel meta-analysis. *Clin Psychol Rev* **85**, 102002 (2021). https://doi.org:10.1016/j.cpr.2021.102002

7 Kendrick, T. *et al.* Routine use of patient reported outcome measures (PROMs) for improving treatment of common mental health disorders in adults. *Cochrane Database Syst Rev* **7**, Cd011119 (2016). https://doi.org:10.1002/14651858.CD011119.pub2

8 Lambert, M. J., Whipple, J. L. & Kleinstäuber, M. Collecting and delivering progress feedback: A meta-analysis of routine outcome monitoring. *Psychotherapy (Chic)* **55**, 520-537 (2018). https://doi.org:10.1037/pst0000167

9 Hudson, J. L. *et al.* Impact of telephone delivered case-management on the effectiveness of collaborative care for depression and anti-depressant use: A systematic review and meta-regression. *PLoS One* **14**, e0217948 (2019). https://doi.org:10.1371/journal.pone.0217948

10 Rens, E., Glazemakers, I. & Van den Broeck, K. Wachten op psychische hulp. De lengte en beleving van wachttijden in de Vlaamse geestelijke gezondheidszorg. (Staten-Generaal van de Geestelijke Gezondheidszorg - Werkgroep Wachttijden, 2020).

11 Richards, D. A. *et al.* Developing a U.K. protocol for collaborative care: a qualitative study. *Gen Hosp Psychiatry* **28**, 296-305 (2006). https://doi.org:10.1016/j.genhosppsych.2006.03.005

12 Brabyn, S. *et al.* The second Randomised Evaluation of the Effectiveness, cost-effectiveness and Acceptability of Computerised Therapy (REEACT-2) trial: does the provision of telephone support enhance the effectiveness of computer-delivered cognitive behaviour therapy? A randomised controlled trial. *Health Technol Assess* **20**, 1-64 (2016). https://doi.org:10.3310/hta20890

13 Gilbody, S. *et al.* Computerised cognitive behaviour therapy (cCBT) as treatment for depression in primary care (REEACT trial): large scale pragmatic randomised controlled trial. *Bmj* **351**, h5627 (2015). https://doi.org:10.1136/bmj.h5627

14 McKenzie, K., Diston, R. & Murray, K. Which elements of socially prescribed activities most improve wellbeing? *Nursing times* **117**, 3 (2021).

15 Dell'Osso, B. *et al.* How to improve adherence to antidepressant treatments in patients with major depression: a psychoeducational consensus checklist. *Ann Gen Psychiatry* **19**, 61 (2020). https://doi.org:10.1186/s12991-020-00306-2

16 *Leven voorbij depressie*, <https://e.issuu.com/embed.html?d=18_jacb_6201_co-creatie_brochure_depressie_nl_issu&hideIssuuLogo=true&u=issuujanssen> (

17 Valentijn, P., Schepman, S., Opheij, W. & Bruijnzeels, M. Understanding integrated care: a comprehensive conceptual framework based on the integrative functions of primary care. *Int J Integr Care* **13**, e010 (2013). https://doi.org:10.5334/ijic.886

18 Declercq, T. *et al.* Richtlijn voor goede medische praktijkvoering: Depressie bij Volwassenen. (Domus Medica VZW, Antwerpen, 2017).

19 Willems, R. *et al.* (Open Science Framework (OSF) osf.io/p9mrq 2025).

20 Malhi, G. S. & Mann, J. J. Depression. *Lancet* **392**, 2299-2312 (2018). https://doi.org:10.1016/s0140-6736(18)31948-2

21 Kroenke, K., Spitzer, R. L. & Williams, J. B. The PHQ-9: validity of a brief depression severity measure. *J Gen Intern Med* **16**, 606-613 (2001). https://doi.org:10.1046/j.1525-1497.2001.016009606.x

22 Levis, B. *et al.* Accuracy of the PHQ-2 Alone and in Combination With the PHQ-9 for Screening to Detect Major Depression: Systematic Review and Meta-analysis. *Jama* **323**, 2290-2300 (2020). https://doi.org:10.1001/jama.2020.6504

23 ELZ-Mechelen-Katelijne. Omgevingsanalyse 2022. Eerstelijnszone Mechelen-Katelijne [Analysis of environment 2022. PCZ Mechelen-Katelijne]. (2022).

24 ELZ-Voorkempen. Omgevingsanalyse Eerstelijnszone Voorkempen, versie mei 2023 [Analysis of environment of PCZ Voorkempen, version May 2023]. (2023).

25 de Jong, K. *et al.* The Outcome Questionnaire (OQ-45) in a Dutch Population: A Cross-Cultural Validation. *Clinical Psychology and Psychotherapy* **14**, 14 (2007).

26 Timman, R., de Jong, K. & de Neve-Enthoven, N. Cut-off Scores and Clinical Change Indices for the Dutch Outcome Questionnaire (OQ-45) in a Large Sample of Normal and Several Psychotherapeutic Populations. *Clin Psychol Psychother* **24**, 72-81 (2017). https://doi.org:10.1002/cpp.1979

27 Arroll, B. *et al.* Validation of PHQ-2 and PHQ-9 to screen for major depression in the primary care population. *Ann Fam Med* **8**, 348-353 (2010). https://doi.org:10.1370/afm.1139

28 Costantini, L. *et al.* Screening for depression in primary care with Patient Health Questionnaire-9 (PHQ-9): A systematic review. *J Affect Disord* **279**, 473-483 (2021). https://doi.org:10.1016/j.jad.2020.09.131

29 Kroenke, K. & Spitzer, R. L. The PHQ-9: A new depression and diagnostic severity measure. *Psychiatric Annals* **32**, 509-521 (2002).

30 Sorensen, K. *HLS-EU-Q Tools and Introduction.* . (2012).

31 Sørensen, K. *et al.* Measuring health literacy in populations: illuminating the design and development process of the European Health Literacy Survey Questionnaire (HLS-EU-Q). *BMC Public Health* **13**, 948 (2013). https://doi.org:10.1186/1471-2458-13-948

32 Grol, R. & Wensing, M. Patients evaluate general/family practice - The EUROPEP Instrument. (2000).

33 *EuroQol Research Foundation. EQ-5D-5L User Guide* <https://euroqol.org/publications/user-guides> (2019).

34 Morisky, D., Ang, A., Krousel-Wood, M. & Ward, H. Predictive validity of a medication adherence measure in an outpatient setting. *J Clin Hypertens (Greenwich)* **10**, 348-354 (2008). https://doi.org:10.1111/j.1751-7176.2008.07572.x. PMID: 18453793

35 Morisky DE, Green LW & DM., L. Concurrent and predictive validity of a self-reported measure of medication adherence. . *Medical Care* **24**, 67-74 (1986).

36 Retraction Statement: Predictive validity of a medication adherence measure in an outpatient setting. *J Clin Hypertens (Greenwich)* **25**, 889 (2023). https://doi.org:10.1111/jch.14718

37 Cameron, K. A. *et al.* Measuring patients' self-efficacy in understanding and using prescription medication. *Patient Educ Couns* **80**, 372-376 (2010). https://doi.org:10.1016/j.pec.2010.06.029

38 iMTA. *iMTA Productivity and Health Research Group. Manual iMTA Medical Cost Questionnaire (iMCQ).* <https://www.imta.nl/questionnaires/imcq/> (2018).

39 Finch, T. L. *et al.* NoMad: Implementation measure based on Normalization Process Theory. [Measurement instrument]. (2015).

40 Braun, V. & Clarke, V. Using thematic analysis in psychology. *Qual Res Psychol*, 77-101 (2006).

41 Holloway, I. & Galvin, K. *Qualitative Research in Nursing and Healthcare. 4th Edition*. (John Wiley & Sons Inc., 2016).
